# Genomic and Chemical Diversity of Bacillus subtilis Secondary Metabolites against Plant Pathogenic Fungi

**DOI:** 10.1128/mSystems.00770-20

**Published:** 2021-02-23

**Authors:** Heiko T. Kiesewalter, Carlos N. Lozano-Andrade, Mario Wibowo, Mikael L. Strube, Gergely Maróti, Dan Snyder, Tue Sparholt Jørgensen, Thomas O. Larsen, Vaughn S. Cooper, Tilmann Weber, Ákos T. Kovács

**Affiliations:** a Bacterial Interactions and Evolution Group, DTU Bioengineering, Technical University of Denmark, Kongens Lyngby, Denmark; b Natural Product Discovery Group, DTU Bioengineering, Technical University of Denmark, Kongens Lyngby, Denmark; c Bacterial Ecophysiology and Biotechnology Group, DTU Bioengineering, Technical University of Denmark, Kongens Lyngby, Denmark; d Institute of Plant Biology, Biological Research Center of the Hungarian Academy of Sciences, Szeged, Hungary; e Microbial Genome Sequencing Center, Pittsburgh, Pennsylvania, USA; f The Novo Nordisk Foundation Center for Biosustainability, Technical University of Denmark, Kongens Lyngby, Denmark; g Department of Microbiology and Molecular Genetics, University of Pittsburgh, Pittsburgh, Pennsylvania, USA; University of California, Berkeley; Technische Universität Berlin

**Keywords:** *Bacillus subtilis*, secondary metabolites, fungal inhibition, antiSMASH, biosynthetic gene clusters, chemodiversity

## Abstract

Bacillus subtilis produces a wide range of secondary metabolites providing diverse plant growth-promoting and biocontrol abilities. These secondary metabolites include nonribosomal peptides with strong antimicrobial properties, causing either cell lysis, pore formation in fungal membranes, inhibition of certain enzymes, or bacterial protein synthesis. However, the natural products of B. subtilis are mostly studied either in laboratory strains or in individual isolates, and therefore, a comparative overview of secondary metabolites from various environmental B. subtilis strains is missing. In this study, we isolated 23 B. subtilis strains from 11 sampling sites, compared the fungal inhibition profiles of wild types and their nonribosomal peptide mutants, followed the production of targeted lipopeptides, and determined the complete genomes of 13 soil isolates. We discovered that nonribosomal peptide production varied among B. subtilis strains coisolated from the same soil samples. *In vitro* antagonism assays revealed that biocontrol properties depend on the targeted plant pathogenic fungus and the tested B. subtilis isolate. While plipastatin alone is sufficient to inhibit *Fusarium* spp., a combination of plipastatin and surfactin is required to hinder growth of Botrytis cinerea. Detailed genomic analysis revealed that altered nonribosomal peptide production profiles in specific isolates are due to missing core genes, nonsense mutation, or potentially altered gene regulation. Our study combines microbiological antagonism assays with chemical nonribosomal peptide detection and biosynthetic gene cluster predictions in diverse B. subtilis soil isolates to provide a broader overview of the secondary metabolite chemodiversity of B. subtilis.

**IMPORTANCE** Secondary or specialized metabolites with antimicrobial activities define the biocontrol properties of microorganisms. Members of the *Bacillus* genus produce a plethora of secondary metabolites, of which nonribosomally produced lipopeptides in particular display strong antifungal activity. To facilitate the prediction of the biocontrol potential of new Bacillus subtilis isolates, we have explored the *in vitro* antifungal inhibitory profiles of recent B. subtilis isolates, combined with analytical natural product chemistry, mutational analysis, and detailed genome analysis of biosynthetic gene clusters. Such a comparative analysis helped to explain why selected B. subtilis isolates lack the production of certain secondary metabolites.

## INTRODUCTION

The rhizosphere is well known as a microbial hot spot since it can be seen as a nutrient-rich oasis surrounded by otherwise nutrient-limited soil regions. This ecosystem comprises a plethora of intra- and interspecies interactions between bacteria, fungi, plants, and higher organisms mediated by a diversity of natural products. In general, soil bacteria are capable of producing a considerable amount of different secondary or specialized metabolites, which, although not essential for growth, might have miscellaneous functions. However, our understanding of the true ecological role of these specialized metabolites has just begun to unfold. On the one hand, secondary metabolites are assumed to be mainly biological weapons that provide the producer strains a competitive advantage in asserting themselves in an ecological niche (1). On the other hand, at subinhibitory concentrations, secondary metabolites are also described as signaling molecules within microbial communities (2, 3), as influencers of cellular differentiation (4), or as molecules alternating the nutrient uptake leading to a reduced niche overlap of competing organisms (5).

One of the most intensely studied species of soil bacteria is Bacillus subtilis, which serves as a laboratory model organism for biofilm formation and sporulation (6). B. subtilis is the type species of the B. subtilis species complex, containing the four original phylogenetically and phenetically homogeneous species B. subtilis, Bacillus amyloliquefaciens, Bacillus licheniformis, and Bacillus pumilus. This species complex was over time complemented with novel species such as Bacillus atrophaeus, Bacillus mojavensis, Bacillus vallismortis, Bacillus tequilensis, Bacillus velezensis, and Bacillus nakamurai, among others (7). Several studies have shown that members of the genus *Bacillus* produce various secondary metabolites, of which many have bioactive properties (8, 9). These secondary metabolites, including polyketides, terpenes, siderophores, and ribosomally and nonribosomally synthesized peptides, are encoded by large biosynthetic gene clusters (BGCs) (10). While numerous natural products have been identified in the B. subtilis species complex, the diversity of secondary metabolite production in numerous isolates from the same niche has not been explored to understand their ecological functions.

Furthermore, it has been shown that *Bacillus* spp. have excellent biocontrol properties by promoting plant growth and reducing plant diseases caused by both plant pathogenic fungi and bacteria (11). These properties are mostly linked to their secondary metabolite profiles. One very potent chemical group of secondary metabolites are nonribosomally synthesized lipopeptides, which have various antimicrobial properties. *Bacillus* spp. produce different lipopeptide isoforms belonging to the families of surfactins, fengycins, and iturins (12). A comparative study of distinct *Bacillus* genomes assigned 11 predicted BGCs to B. subtilis strains (13). Notably, a predicted BGC is not proof of the synthesis of the natural product. Gene silencing or the absence of unidentified environmental triggers can be a reason for the lack of BGC expression (10).

This study focused on nonribosomal peptides (NRPs) produced by recently obtained B. subtilis soil isolates, whose biosyntheses depend on the phosphopantetheinyl transferase Sfp. This transferase plays an essential role in the NRP syntheses in B. subtilis since it functions as an activator of the peptidyl carrier protein domains, converting them from the inactive apo-form to the active holo-form by transferring the 4-phosphopantetheine of coenzyme A as a prosthetic group to a conserved serine residue (14). NRPs are synthesized by large enzyme complexes, nonribosomal peptide synthetases (NRPSs) (10). B. subtilis harbors three NRPS gene clusters (surfactin, plipastatin, and bacillibactin) and one hybrid nonribosomal peptide synthetase-polyketide synthase (NRPS-PKS) gene cluster (bacillaene). The domesticated B. subtilis laboratory strain 168 contains an inactive *sfp* gene due to a frameshift mutation, causing an incapability of NRP production (14–16). Surfactin, encoded by the *srfAA-srfAD* gene cluster, is a well-studied and multifunctional secondary metabolite. The biosurfactant reduces surface tension needed for swarming and sliding motility (17, 18). Surfactin’s cytolytic activity is mainly based on its surfactant activity, causing cell lysis due to penetration of bacterial lipid bilayer membranes and forming ion-conducting channels (19–21). Studies revealed that surfactin displays bioactivity against Listeria monocytogenes and different *Legionella* spp. *in vitro* and at low concentrations and damages the membrane of Staphylococcus aureus (22–24). It was recently discovered that surfactin increases the availability of oxygen for B. subtilis in liquid cultures and eases the exploitation of nonpreferred carbon sources in *B. amyloliquefaciens* (25, 26). The powerful antifungal lipopeptide plipastatin, chemically very similar to fengycin but with a different d-tyrosine position within the peptide backbone, is synthesized by the *ppsA*-*ppsE* gene cluster. Recently, it has been shown that the plipastatin BGC is present in the B. subtilis clade, while the fengycin BGC is found in the *B. amyloliquefaciens* and *B. velezensis* clades (27). The detailed mode of action of plipastatin is not yet unraveled, but it is believed that it functions as an inhibitor of phospholipase A2, forming pores and causing morphological changes in the fungal membrane and cell wall (10, 28, 29). Many studies have shown that plipastatin and fengycin are bioactive against diverse filamentous fungi (24, 30–35). Bacillaene, expressed from the *pksB-pksS* gene cluster, is a broad-spectrum antibiotic mainly acting by inhibiting bacterial protein synthesis; additionally, it was also shown that it could protect cells and spores from bacterial predators (36, 37). Bacillibactin, synthesized by the *dhbACEBF* gene cluster, is a siderophore and transports iron from the environment to the cell (38). However, no studies have been published on its direct antimicrobial properties.

Most studies in the literature concentrate on single *Bacillus* species isolates, which are often selected due to their excellent antimicrobial properties. In this study, we endeavored a comprehensive overview of the chemodiversity within the B. subtilis species. Therefore, a special focus was placed on recently and partly coisolated B. subtilis environmental strains without prior bioactivity screening. We concentrated on differences in their antifungal properties, NRP production, the genomic background of secondary metabolite arsenal, and intraspecies interactions. Antagonism assays tested the antifungal properties of natural isolates and their respective NRP mutant derivatives against the three plant pathogenic fungi Fusarium oxysporum, Fusarium graminearum, and Botrytis cinerea. F. oxysporum is a known plant pathogenic fungus causing *Fusarium* wilt in tomato, tobacco, or banana plants, among others (39). F. graminearum causes *Fusarium* head blight in different cereal crops (40). *B. cinerea* has a very wide variety of hosts. However, its main hosts are wine grapes and other fruits, in which it causes gray mold disease (41, 42). Using a B. subtilis isolate library, we identified the NRPs responsible for inhibiting two *Fusarium* spp. and *Botrytis cinerea*. Further, using fungal inhibition profiles, chemical detection of the NRPs, and detailed genomic analysis, we discovered that isolates originating from the same soil sample site possess distinct secondary metabolite production abilities, suggesting chemical differentiation of B. subtilis in the environment. The findings of intraspecies interactions among isolates coinhabiting close microenvironments suggest an impact of accessory BGCs on their inhibition potential and secondary metabolite susceptibility.

## RESULTS

### Antifungal potential of B. subtilis isolates.

**FIG 1:**
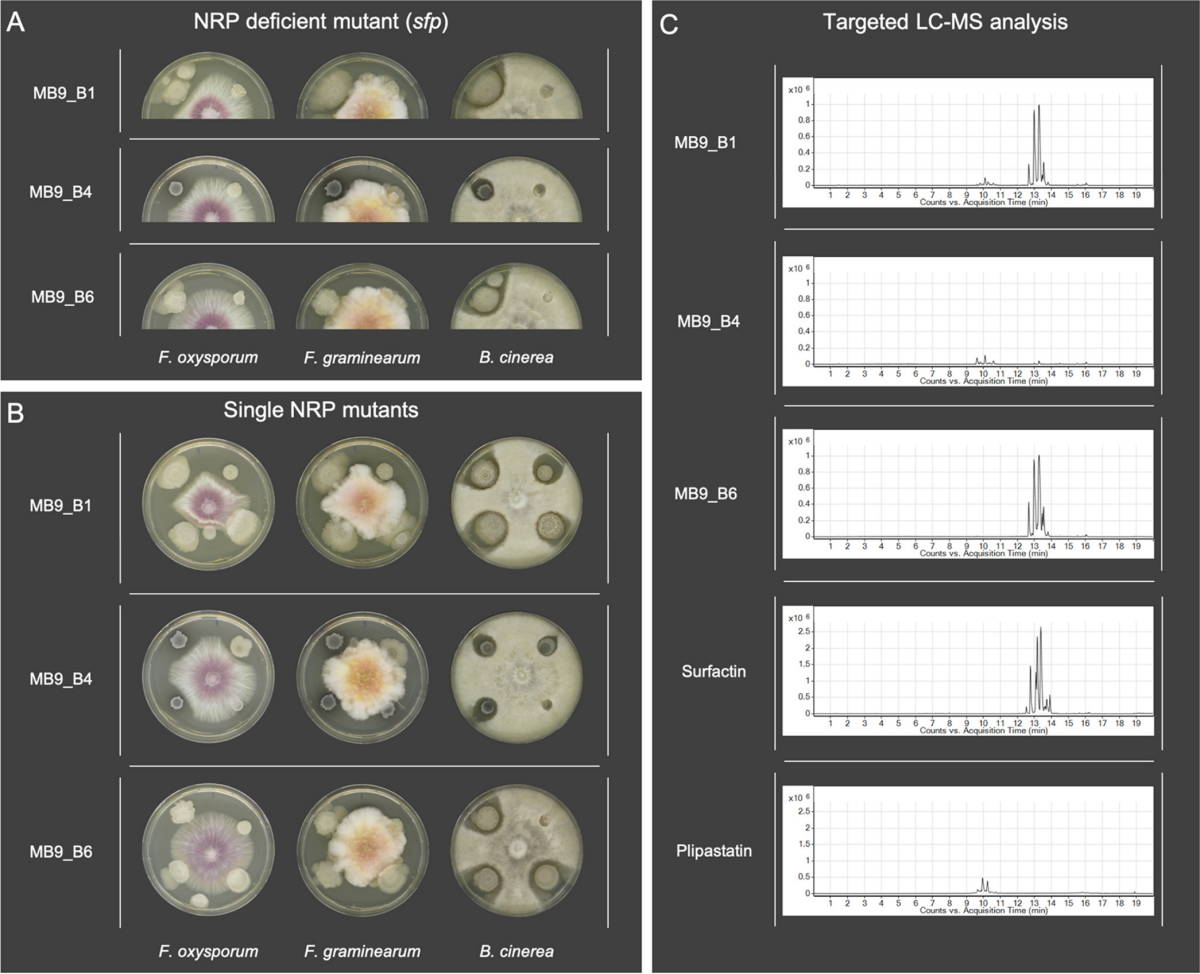
(A) Antagonism assays between the plant pathogenic fungi Fusarium oxysporum, Fusarium graminearum, and *Botrytis cinerea* and the B. subtilis soil isolates (left) as well as their NRP-deficient *sfp* mutants (right). (B) Antagonism assays between the plant pathogenic fungi and B. subtilis soil isolates (upper left) as well as their single nonribosomal peptide *srfAC* (upper right, no surfactin), Δ*ppsC* (lower right, no plipastatin), and Δ*pksL* (lower left, no bacillaene) mutants. A 5-μl quantity of bacterial overnight culture and fungal spore suspension was spotted onto the edges (bacteria) and in the center (fungi) of potato dextrose agar (PDA) plates. Strains were cocultivated at 21 to 23°C for 6 days. (C) Extracted ion chromatograms (*m/z* 1,000 to 1,600) display various levels of production of surfactin and plipastatin among B. subtilis soil isolates. The standard mixtures of plipastatin and surfactin are shown at the bottom. Multiple peaks in the LC-MS traces among the isolates and standards show different surfactin and plipastatin analogs with different fatty acid substitutions. The presence of surfactin and plipastatin in the isolates’ extracts was confirmed by retention time comparisons with the standards and by tandem mass spectrometry (MS/MS) fragmentation studies.

A library of B. subtilis isolates has been established from various locations in Denmark and Germany (see Materials and Methods). To confirm that NRPs produced by these B. subtilis soil isolates have antifungal potential, we screened both wild-type (WT) isolates and their *sfp* mutants (Fig. 1A) as well as their *srfAC*, Δ*ppsC*, and Δ*pksL* single NRP mutant derivatives (Fig. 1B) against the three plant pathogenic fungal strains F. oxysporum, F. graminearum, and *B. cinerea*.

The mutant screen allowed us to verify if a single NRP or a mixture of them is responsible for the bioactivity.

**FIG 2:**
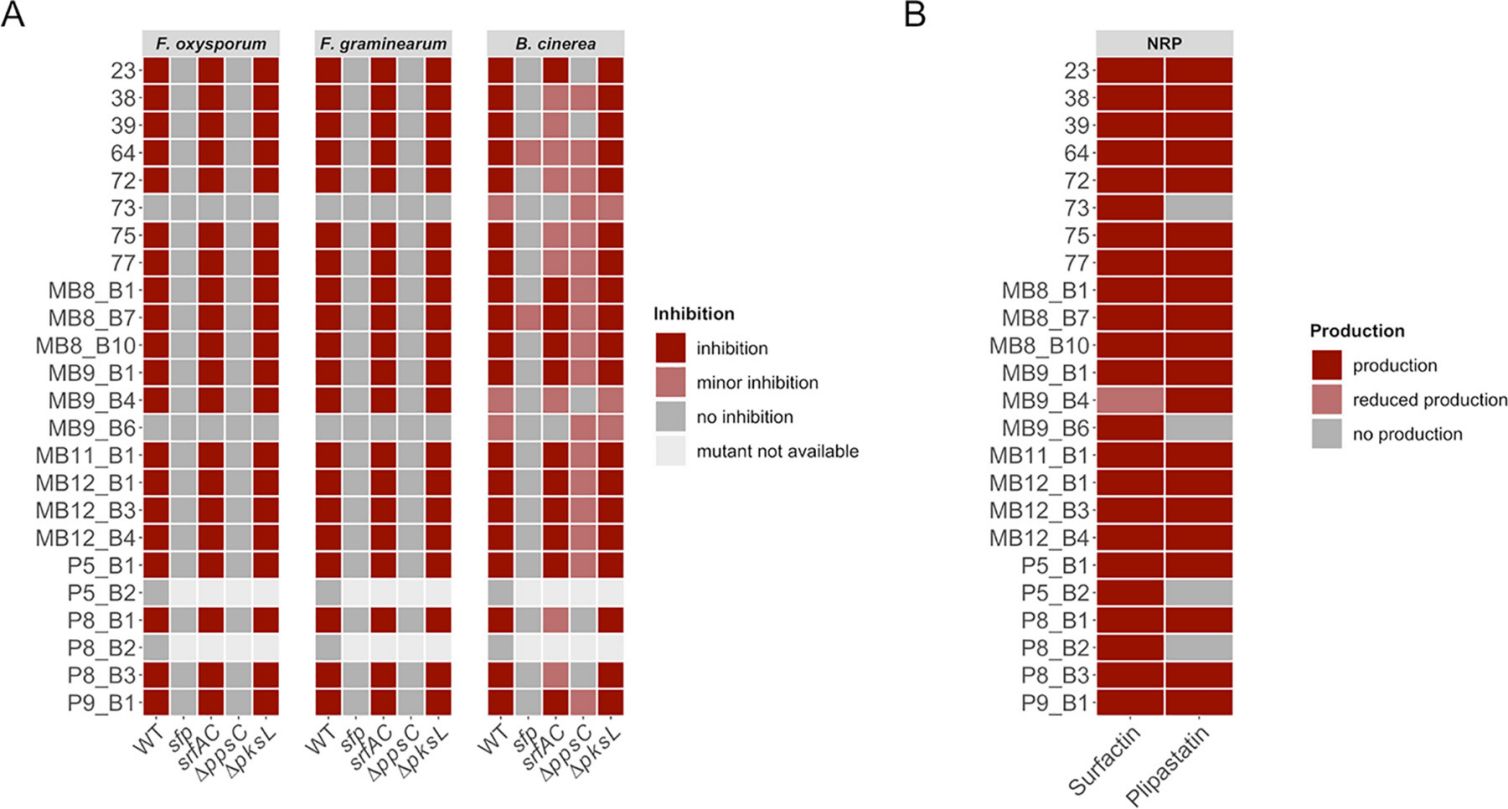
(A) Overview of qualitative evaluation of antagonisms assays assigned to inhibition, minor inhibition, and no inhibition. Strains P5_B2 and P8_B2 were not naturally competent, and no NRP mutants could be created. (B) Overview of NRP production of wild-type soil isolates based on the detection of surfactin and plipastatin in the extracts by ESI-MS. The production of the compounds was classified as production (detected), reduced production (detected but at a lower level), and no production (undetected).

The qualitative assessment of antifungal potential from 24 tested isolates was classified into inhibition, minor inhibition, and no inhibition by comparing mutant strains to their respective wild types and comparing wild types with each other (Fig. 2A; see also Fig. S2 in the supplemental material). The incidence of a distinct inhibition zone was defined as inhibition, while “no inhibition” refers to a total loss of inhibitory potential, which appeared in bacterial colonies surrounded or overgrown by the fungus.

To increase the possibility of differentiating between slight distinctions, we assigned strains exhibiting a reduced antagonism to the class minor inhibition. This observation differed between mutant derivatives and WTs. We specified minor inhibitions for WT, *srfAC*, Δ*ppsC*, and Δ*pksL* strains when a thin layer of fungal hyphae was growing into the visible clearing zone toward the bacterial colony. In contrast, for *sfp* mutants, it described a not-entire loss of bioactivity (Fig. S1). The two isolates B. subtilis P5_B2 and B. licheniformis P8_B2 were not naturally competent. Thus, we were unable to create NRP mutants. However, both wild types showed no inhibition of *Fusarium* spp. or *B. cinerea*.

10.1128/mSystems.00770-20.1 FIG S1 The three evaluation classes, inhibition, minor inhibition, and no inhibition, in the screening against *B. cinerea* for each B. subtilis genotype. The absence of a picture symbolizes the absence of the specific observation in the genotype. Minor inhibition was assigned to screening results if the fungus was inhibited, but thinner hyphae grew towards the bacterial colony. Download FIG S1, TIF file, 2.2 MB. Copyright © 2021 Kiesewalter et al. 2021 Kiesewalter et al. https://creativecommons.org/licenses/by/4.0/ This content is distributed under the terms of the Creative Commons Attribution 4.0 International license.

10.1128/mSystems.00770-20.2 FIG S2 Antagonism assays between the plant pathogenic fungi F. oxysporum, F. graminearum, and *B. cinerea* and the B. subtilis soil isolates as well as their NRP-deficient *sfp* mutants (left column), single NRP mutants (middle column; *srfAC* [top right], Δ*ppsC* [bottom right], Δ*pksL* [bottom left]) and surfactin-plipastatin double mutants (right column, bottom left). The antagonism assays with B. subtilis 3610 are shown at the bottom. Strains were spotted as shown in the schemes on PDA plates and incubated at 21 to 23°C for 6 days. Download FIG S2, TIF file, 2.6 MB. Copyright © 2021 Kiesewalter et al. 2021 Kiesewalter et al. https://creativecommons.org/licenses/by/4.0/ This content is distributed under the terms of the Creative Commons Attribution 4.0 International license.

Twenty of 24 tested wild types showed inhibition of F. oxysporum and F. graminearum, whereas their *sfp* mutants showed no growth inhibition. Exceptions were strains 73 and MB9_B6, which showed no antagonistic effects against *Fusarium*. For all 20 bioactive strains, only their Δ*ppsC* mutants, incapable of producing plipastatin, lost the bioactivity against both *Fusarium* species, similar to their *sfp* mutants.

Additionally, the screening revealed that *B. cinerea* is not as sensitive to a single compound as the tested *Fusarium* strains (Fig. 2A). All tested WT strains inhibited *B. cinerea*, while three strains (73, MB9_B4, and MB9_B6) showed minor inhibition. Similar to the *Fusarium* antagonism, the bioactivity against *Botrytis* was *sfp* dependent for most of the tested strains. However, two of the *sfp* mutants (64 and MB8_B7) maintained antagonistic properties, albeit reduced compared to their respective WTs. The inactivation of either plipastatin or surfactin production caused different screening results depending on the specific soil isolate.

Four strains (23, 39, P8_B1, and P8_B3) showed a clear plipastatin-dependent bioactivity, since a total loss of inhibition was observable when the plipastatin BGC was disrupted in these strains. Furthermore, 10 strains (MB8_B1, MB8_B7, MB8_B10, MB9_B1, MB11_B1, MB12_B1, MB12_B3, MB12_B4, P5_B1, and P9_B1) indicated a partial plipastatin-dependent antagonism, where the *pps* BGC disruption led to a reduced inhibition potential but not a complete loss of bioactivity. In addition, four strains (38, 72, 75, and 77) demonstrated that both plipastatin and surfactin impact the bioactivity. In these strains, the absence of either one of them reduced their antifungal potential. Interestingly, two strains (73 and MB9_B6) showed no inhibition of *B. cinerea* when their surfactin production was disturbed, and no differences between the Δ*ppsC* mutants and their wild-type strains could be observed. In contrast, strain MB9_B4 lost its ability to inhibit *B. cinerea* when plipastatin was not produced, whereas the absence of surfactin production did not influence its inhibition capability. Furthermore, in eight strains (38, 39, 64, 72, 75, 77, P8_B1, and P8_B3), disruption of surfactin production led to reduced bioactivity. However, none of the bacillaene (Δ*pksL*) mutants displayed changes in the antifungal potential compared to their WTs.

In conclusion, the screening demonstrated that plipastatin is the only compound responsible for the inhibition of F. oxysporum and F. graminearum. Moreover, the primarily *sfp*-dependent bioactivity against B. *cinerea* is either plipastatin, partially plipastatin, or both plipastatin and surfactin dependent. However, among all strains, three stood out in the antifungal screening. Strains 73 and MB9_B6 lacked inhibition of F. oxysporum and F. graminearum and displayed reduced inhibition of *B. cinerea*. Both their *sfp* and *srfAC* derivatives showed a complete loss of bioactivity against *B. cinerea*. In contrast to these strains, isolate MB9_B4 showed no inhibition of *B. cinerea* when the plipastatin BGC was disrupted.

### Chemical characterization of B. subtilis isolates and their mutant derivatives.

Screening of antifungal activities revealed potential differences in surfactin and plipastatin production among the isolates. Therefore, to compare the qualitative production of these NRPs among the soil isolates, a targeted liquid chromatography-mass spectrometry (LC-MS) analysis was performed targeting compounds with *m/z* values between 1,000 and 1,600 (43, 44). Interestingly, even coisolated strains showed various degrees of NRP production (Fig. 1C). The qualitative analysis disclosed that the majority of strains produced both surfactin and plipastatin (Fig. 2B and Fig. S3), while the three peculiar strains from the antifungal screening (73, MB9_B4, and MB9_B6) and the two nontransformable strains (P5_B2 and P8_B2) had a distinct natural product profile. Plipastatin was not detectable in the extracts from strains 73, MB9_B6, P5_B2, and P8_B2. The absence of plipastatin production in these isolates correlates with the lack of antagonism against the *Fusarium* species and either reduced or lost *B. cinerea* inhibition compared to other isolates. Additionally, strain MB9_B4, with a strongly lowered surfactin production level, displayed reduced *B. cinerea* inhibition. Notably, the plipastatin mutant of MB9_B4 exhibited a total loss of antagonism. The results demonstrate that if wild types do not produce plipastatin or surfactin and the production of the counterpart NRP is genetically hindered, strains lose the bioactivity against *B. cinerea*.

10.1128/mSystems.00770-20.3 FIG S3 Extracted ion chromatograms (*m/z* 1,000 to 1,600) show the presence of surfactin and plipastatin analogs produced by recently isolated B. subtilis strains grown on PDB agar medium for 3 days. Surfactin was detected in strain MB9_B4, but its quantity was lower than in other isolates. Plipastatin was not detectable in extracts of strains 73, MB9_B6, and P5_B2 and B. licheniformis strain P8_B2. Surfactins and fengycins are all in the *m/z* range 1,000 to 1,600, which can be detected by ESI-MS. Multiple peaks in the LC-MS traces among the isolates and standards show different surfactin and plipastatin analogs with different fatty acids substitutions. The presence of surfactin and plipastatin in the isolates’ extracts was confirmed by retention time comparisons with the standards and by MS/MS fragmentation studies. Download FIG S3, TIF file, 1.8 MB. Copyright © 2021 Kiesewalter et al. 2021 Kiesewalter et al. https://creativecommons.org/licenses/by/4.0/ This content is distributed under the terms of the Creative Commons Attribution 4.0 International license.

### Impact of both plipastatin and surfactin on inhibition of *B. cinerea*.

In combination with chemical profiling, the deletion mutant screen suggested that for most B. subtilis strains, plipastatin and surfactin are primarily responsible for the suppression of *B. cinerea*. The absence of plipastatin led to a complete loss or reduction of bioactivity in 17 strains, while the absence of surfactin caused a reduced inhibition in 7 strains. A necessity for both NRPs for full anti-*B. cinerea* bioactivity was strengthened by the screening results for the naturally impaired NRP producer strains 73, MB9_B4, and MB9_B6.

To test this hypothesis, both BGCs were disrupted in strains found to produce both compounds detected by chemical profiling. However, all three tested *srfAC*-Δ*ppsC* double mutants (75, MB8_B1, and MB9_B1) maintained bioactivity against the fungus (Fig. S4A), even though targeted LC-MS analysis of two of these tested strains confirmed the lack of both lipopeptides (Fig. S4C). These data indicate that the bioactivity is for some of the strains not caused by only surfactin and plipastatin. The *sfp*-dependent NRPs bacillaene and bacillibactin might contribute to a smaller extent to the inhibition of *B. cinerea* growth.

10.1128/mSystems.00770-20.4 FIG S4 (A) Antagonism assays between *Botrytis cinerea* and B. subtilis wild types (upper left) as well as their *srfAC* (upper right), Δ*ppsC* (lower right), and *srfAC*-Δ*ppsC* (lower left) mutants. (B) Antagonism assays between *Botrytis cinerea* and B. subtilis 3610 as well as its NRP mutants. (Left plate) WT (upper left) and *srfAC* (upper right), Δ*ppsC* (lower right), and Δ*pksL*(lower left) mutants. (Right plate) WT (upper left) and *sfp* (upper right), *srfAC*-Δ*ppsC* (lower right), and *srfAC*-Δ*ppsC*-Δ*pksL* (lower left) mutants. Strains were cocultivated on PDA plates and incubated at 21 to 23°C for 6 days. (C) Extracted ion chromatograms (*m/z* 1,000 to 1,600) of B. subtilis mutants of the strains MB8_B1 and P8_B1 grown on PDA for 3 days show the presence of surfactin and plipastatin analogs in the extracts. The chromatograms of the standards of surfactin and plipastatin and the PDA medium are shown at the bottom. Download FIG S4, TIF file, 1.1 MB. Copyright © 2021 Kiesewalter et al. 2021 Kiesewalter et al. https://creativecommons.org/licenses/by/4.0/ This content is distributed under the terms of the Creative Commons Attribution 4.0 International license.

### Prediction of biosynthetic gene cluster potential of the isolates from their genome sequences.

**FIG 3:**
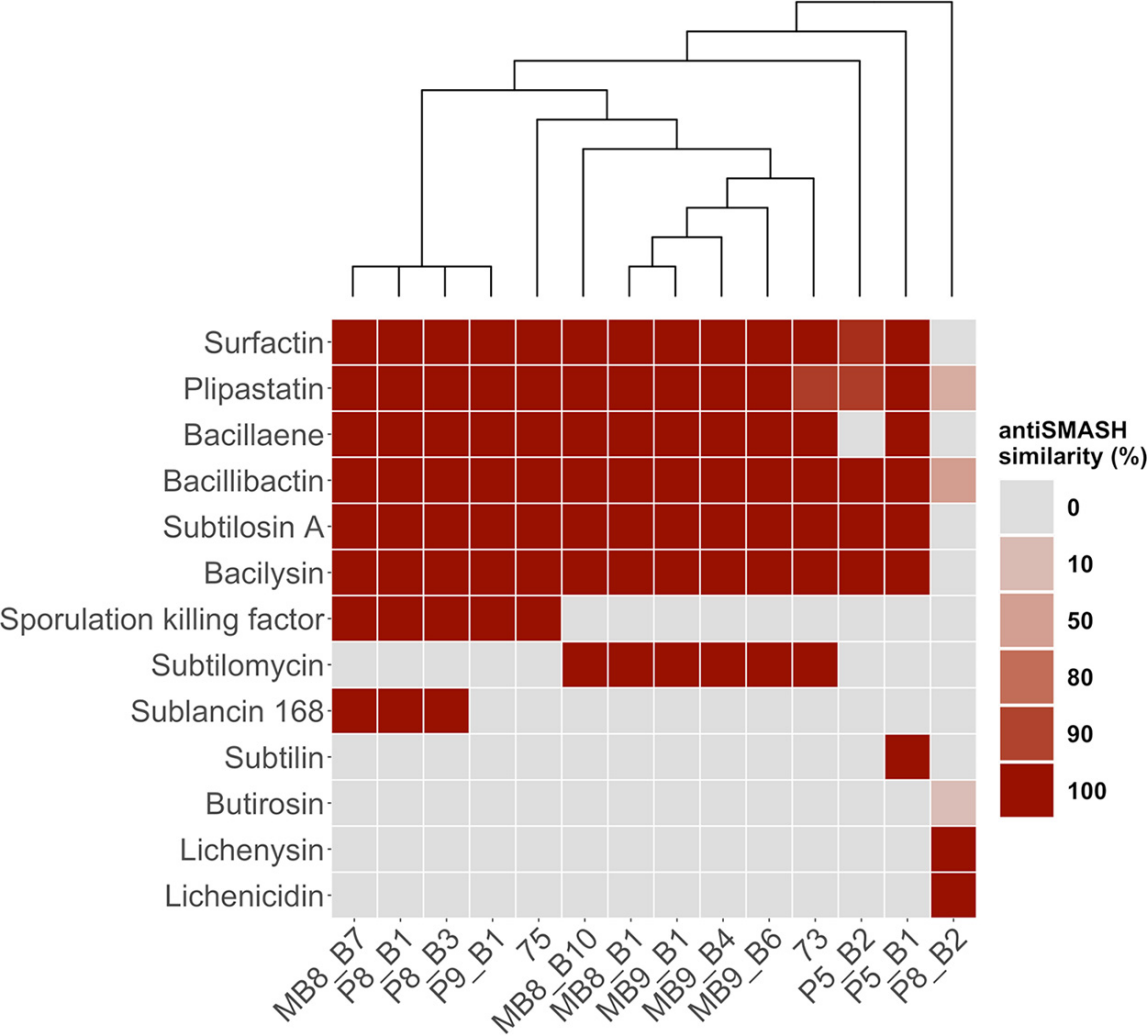
Overview of predicted biosynthetic gene clusters (BGCs) by antiSMASH of 13 B. subtilis and 1 B. licheniformis (right) soil isolate. The color code visualizes the similarity of BGCs to a reference BGC, whereby the gray color (0%) indicates their absence. The cladogram is based on a core gene alignment by the pan-genome pipeline Roary.

Based on the antifungal screening results and the origin of the isolate, 13 B. subtilis strains were selected for genome sequencing (45). Additionally, we sequenced the genome of the closely related B. licheniformis strain P8_B2 to highlight discrepancies of this species from B. subtilis. The genomes were analyzed with antiSMASH (46) to obtain an overview of the predicted BGCs, which have similarities to already known clusters (Fig. 3). Importantly, these predictions highlight the genomic potential but not the actual production of secondary metabolites. Additionally, the whole BGCs and not solely the core genes were compared to gene clusters of appropriate reference strains.

The BGCs for the *sfp*-dependent NRPs surfactin, plipastatin, bacillaene, and bacillibactin were predicted in all B. subtilis isolates except for isolate P5_B2, with no predicted bacillaene BGC. The surfactin gene cluster showed for the majority of strains a similarity of 100% compared to the reference, while only isolate P5_B2 exhibited a lower similarity due to minor differences in genes of the gene cluster. Likewise, for the plipastatin gene cluster, the greater number of B. subtilis strains showed a similarity of 100%, except strains 73 and P5_B2, which both displayed absent genes compared to the reference gene cluster. Bacillibactin, subtilosin A and bacilysin are present in all B. subtilis strains, with a similarity of 100%. The sporulation killing factor is present in five strains, but these lack the gene cluster for subtilomycin production. In contrast, six strains are predicted to code for subtilomycin synthesis and are conversely missing the genes of the sporulation killing factor. Phelan et al. observed that the subtilomycin gene cluster of a marine isolate is present in the genomic locus of the sporulation killing factor gene cluster (47). Finally, neither sporulation killing factor nor subtilomycin gene clusters are predicted to be present in strains P5_B1 and P5_B2. However, subtilin genes seem to be present in P5_B1. Strain P5_B2 represents an outlier among the B. subtilis isolates since it possesses only the BGCs for the core secondary metabolites of B. subtilis, except bacillaene, but none of the accessory BGCs differentially present in the others. Additionally, all 13 B. subtilis strains harbor four unidentified BGCs: two terpene, one type III PKS, and one tRNA-dependent cyclodipeptide synthase BGC.

Interestingly, strain P5_B1 has further predictions for one lanthipeptide and one bacteriocin BGC. In line with the inhibition data, B. licheniformis P8_B2 has a deviating profile of BGCs. Three gene clusters show similarity to plipastatin, bacillibactin, and butirosin, at 30%, 53%, and 7%, respectively. Additionally, the BGCs for the species-specific secondary metabolites, lichenysin and lichenicidin, were predicted in P8_B2 with a 100% similarity.

### Detailed comparison of BGC structures explains the lack of NRP production.

**FIG 4:**
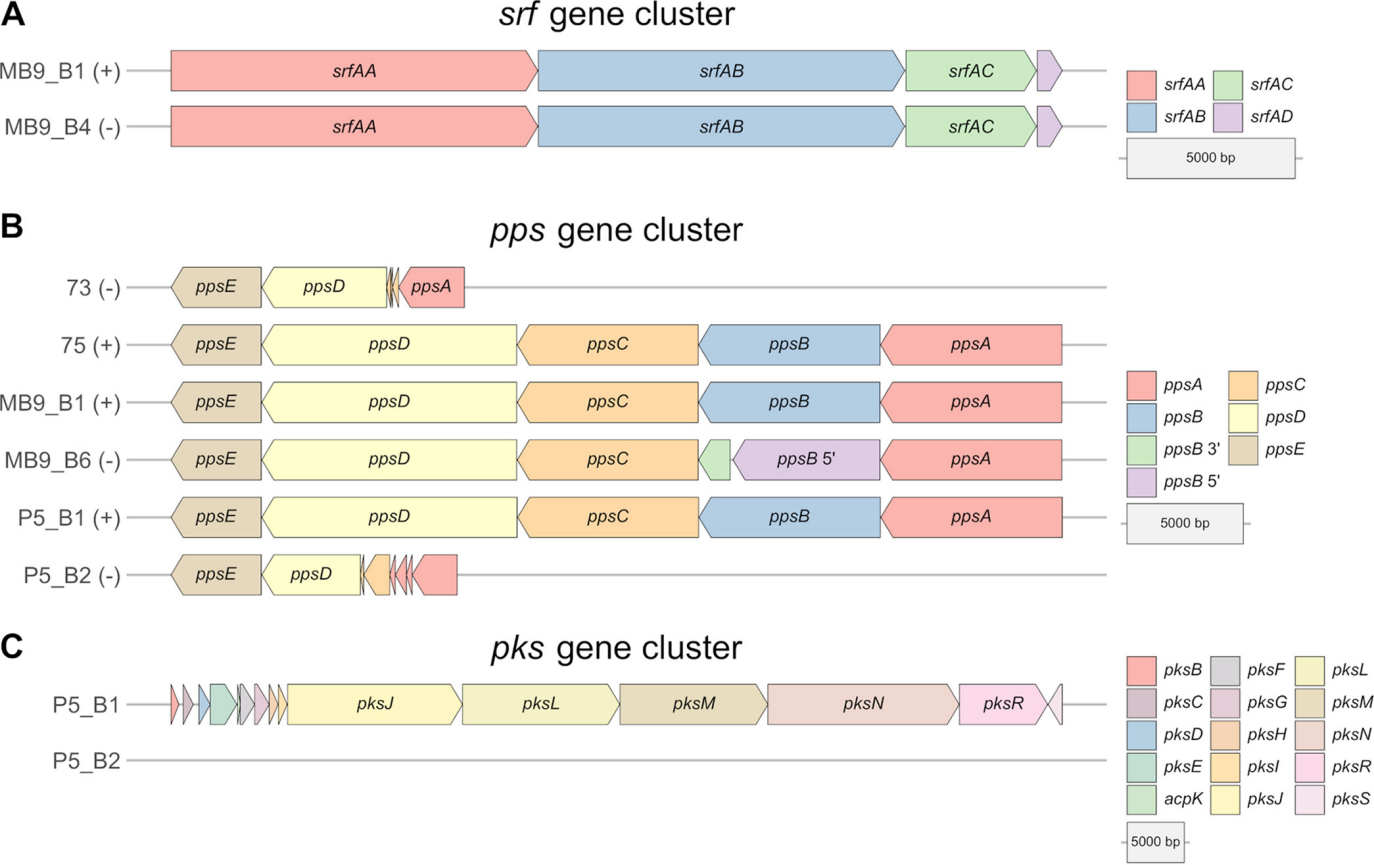
Comparison of core genes of the biosynthetic gene clusters surfactin (A) and plipastatin (B) from coisolated B. subtilis strains, which were classified into producer (+) and nonproducer/production-hampered (−) strains based on the targeted LC-MS analysis. (C) Comparison of core genes of the biosynthetic gene clusters of bacillaene from two coisolated B. subtilis strains.

Differences in both the antifungal potential and plipastatin or surfactin production based on the targeted LC-MS analysis combined with the BGC prediction with antiSMASH led us to concentrate more on the nonproducer or predicted nonproducer strains MB9_B4, 73, MB9_B6, and P5_B2. To understand why these strains show these characteristics, we analyzed the core genes of surfactin, plipastatin, and bacillaene in their presence and absence and compared them to the BGCs of coisolated producer strains (Fig. 4).

MB9_B4 showed a hampered surfactin production, even though all core genes of the surfactin BGC are present equally to the levels in the coisolated producer strain MB9_B1 (Fig. 4A). We further analyzed genes involved in the regulation of surfactin BGC transcription. Comparison of the *comA* genes of all 13 B. subtilis strains, which express the response regulator protein ComA, revealed six mutated regions. However, five of them were silent mutations, but the point mutation at nucleotide position 3 is unique for MB9_B4 and causes an alteration in the translation initiating methionine (Fig. S5B). Consequently, the coding region of *comA* is reduced by 13 amino acids in this strain. It was shown that ComA triggers the transcription of the *srfA* operon directly by binding to its promoter region (48–50). The conserved residues at amino acid positions 8 and 9, reported to be among three targets for ComP-catalyzed phosphorylation, are not translated due to the mutation in MB9_B4 (51). The results led us to assume that surfactin production of MB9_B4 might be hampered due to altered regulatory processes.

10.1128/mSystems.00770-20.5 FIG S5 (A) Strain MB9_B6 harbors a point mutation (6224G to A), causing a point-nonsense mutation in the second domain of the *ppsB* gene due to an amino acid change from tryptophan to a termination codon (W2075X). (B) The point mutation in the *comA* gene of strain MB9_B4 (3G to A) causing a change of the translation initiating methionine. Download FIG S5, TIF file, 0.6 MB. Copyright © 2021 Kiesewalter et al. 2021 Kiesewalter et al. https://creativecommons.org/licenses/by/4.0/ This content is distributed under the terms of the Creative Commons Attribution 4.0 International license.

Plipastatin was not detectable in the strains 73, MB9_B6, and P5_B2, and all were incapable of inhibiting the tested *Fusarium* strains. Analyses of the *pps* gene cluster from strains 73 and P5_B2 revealed only smaller fragments of the genes *ppsA*, *ppsC*, and *ppsD*, a complete absence of *ppsB*, but a present *ppsE* gene (Fig. 4B). Interestingly, MB9_B6 harbors all five *pps* core genes. However, the *ppsB* gene translation is interrupted by a point-nonsense mutation, G→A, which causes an amino acid change from tryptophan to a termination codon (Fig. S5A). The resulting nontranslated region of 41 amino acids leads to a dysfunction of the second domain’s carrier and epimerization regions. Therefore, the plipastatin production is most likely inactive due to either missing core genes or a disrupted *ppsB* gene.

In this study, we did not measure the production of bacillaene. However, the genomic background of strain P5_B2 is missing all core genes of the *pks* gene cluster (Fig. 4C). This observation strongly supports the assumption that P5_B2 is incapable of producing bacillaene.

We conclude that differences in the synthesis of surfactin, plipastatin, and bacillaene are caused by either regulatory processes, a disrupted core gene caused by a point-nonsense mutation, or a loss of several core genes, highlighting the diversity of NRP production in soil isolates of B. subtilis.

### Intraspecies interactions of soil isolates.

**FIG 5:**
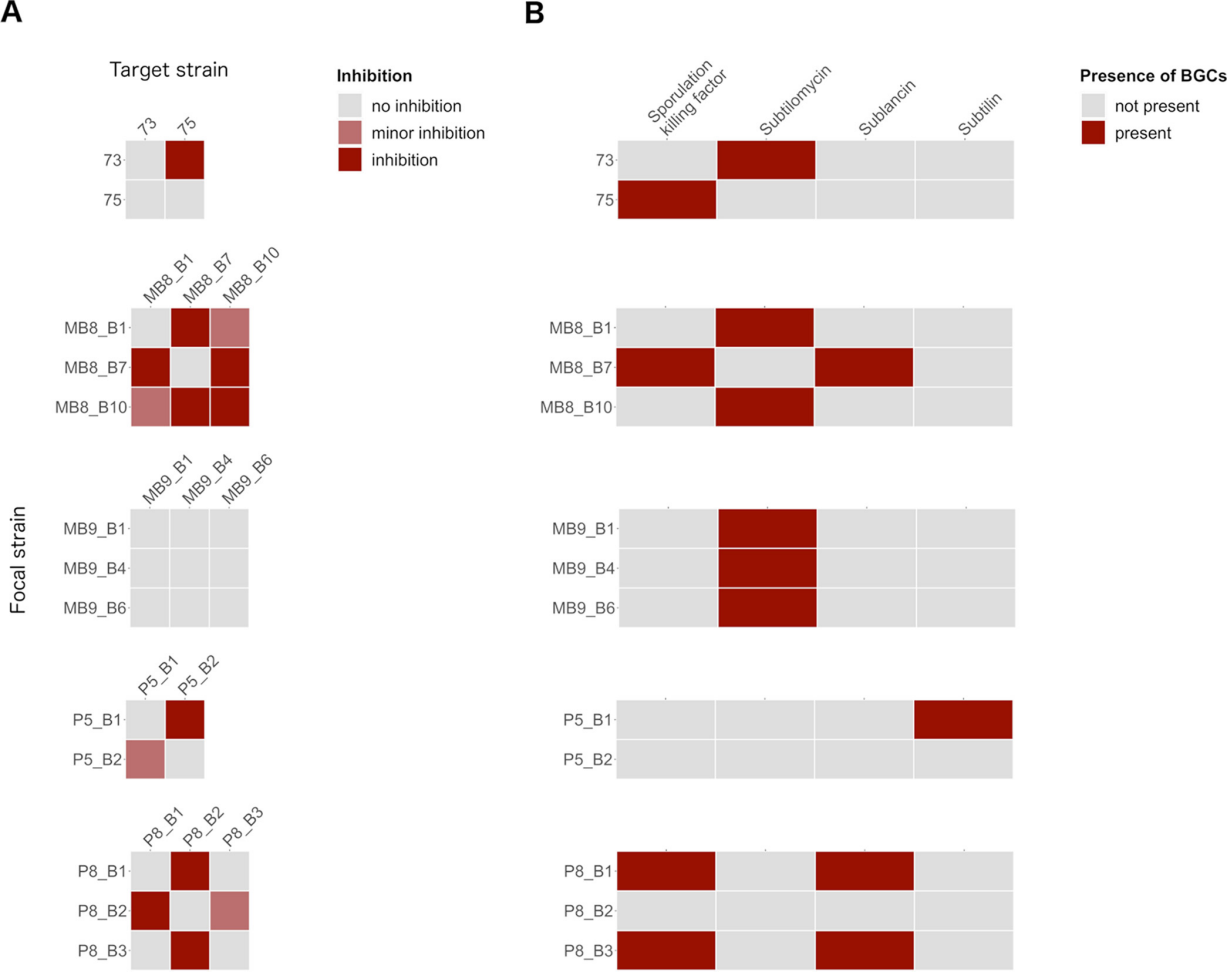
(A) Overview of intraspecies inhibition of coisolated B. subtilis strains. Focal strains were tested for their capability to inhibit each coisolated target strain. The inhibition potential was evaluated by examining the zone surrounding the focal strain colony and classified into inhibition (occurrence of a cell-free zone and growth reduction), minor inhibition (only growth reduction), and no inhibition (neither cell-free zone nor growth reduction). The target strains were embedded in 1% LB agar, and the focal strains (8 μl) were spotted on top. Plates were incubated at 37°C for 24 h. (B) Overview of predicted and known accessory BGCs by antiSMASH.

In addition to the antifungal activities, strains isolated from the same sampling site were cocultivated to determine if they have the capacity to inhibit one another (Fig. 5A). The inhibition results of coisolates were furthermore compared to the predicted accessory BGCs (Fig. 5B). Importantly, none of the strains showed self-inhibition, except MB8_B10, which displayed a clear inhibition zone. Strain 73 inhibited strain 75, but not vice versa. The BGC of the lantibiotic subtilomycin is predicted for strain 73 but absent in strain 75. Strains MB8_B1 and MB8_B10 have a predicted subtilomycin BGC and demonstrated only minor inhibition of each other. However, both strains inhibited MB8_B7, which is lacking this gene cluster. On the other hand, MB8_B7 inhibited both MB8_B1 and MB8_B10, which might be traced back to the predicted BGCs of sublancin or sporulation killing factor. Strains MB9_B1, MB9_B4, and MB9_B6 showed one common BGC, subtilomycin, and in line with this, no inhibition was detectable during the antagonism screens. P5_B1, harboring a predicted subtilin BGC, inhibited P5_B2, which has none of the targeted BGCs predicted. Nevertheless, P5_B2 still showed a reduced inhibition of P5_B1. Strains P8_B1 and P8_B3, harboring a predicted sublancin BGC, inhibited their coinhabitant P8_B2. B. licheniformis strain P8_B2 had none of the targeted BGCs predicted but the B. licheniformis-specific BGCs lichenysin and lichenicidin. Still, P8_B2 inhibited P8_B1 and showed minor inhibition of P8_B3.

Based on the screening results and predicted BGCs, we can hypothesize that strains harboring the same BGCs did not inhibit each other or showed only minor inhibition. Moreover, strains with different BGCs had variable inhibitory effects on each other, possibly due to the lack of resistance genes for the specific secondary metabolite.

## DISCUSSION

Undomesticated isolates of B. subtilis produce a wide range of different secondary metabolites, defining their biocontrol properties. The produced secondary metabolites affect fungal and bacterial growth and differentiation, and possibly other micro- and macroorganisms. Our study provides an overview of the antifungal properties and secondary metabolite profiles of recently and partly coisolated environmental strains of B. subtilis.

Our screening results revealed that antifungal properties vary among B. subtilis soil isolates and, interestingly, among coisolated strains from the same soil sample. We demonstrated that only plipastatin is necessary to inhibit the growth of F. oxysporum and F. graminearum. In contrast, the anti-*Botrytis* potential of B. subtilis is linked to multiple *sfp*-dependent NRPs, of which surfactin and plipastatin contribute the most to full fungal inhibition. Disrupting the BGC responsible for the production of surfactin or plipastatin in the strain that produces only one of these NRPs eliminated the strains’ anti-*Botrytis* activity. In contrast, mutation of both BGCs in a strain that originally produces both surfactin and plipastatin still maintains slight activity against *B. cinerea*, despite a clear *sfp*-dependent inhibition. The impact of bacillaene and bacillibactin on the anti-*Botrytis* activity must be investigated in further studies. The clear *sfp*-dependent antifungal properties for most isolates refuse an effect of the commonly predicted, *sfp*-independent antifungal NRP bacilysin. However, it would be interesting to explore if bacilysin, cell wall-degrading enzymes, or the predicted unknown secondary metabolites are responsible for the remaining but strongly reduced bioactivity of the two *sfp* mutants of strains 64 and MB8_B7 against *B. cinerea*.

A more in-depth analysis and comparison of the produced surfactins and plipastatins of the isolates must be performed to explore possible differences in their compositions and chemical structures impacting the overall bioactivity potential. Nevertheless, the production of plipastatin or a combination of both plipastatin and surfactin is essential in B. subtilis to suppress *B. cinerea* growth.

Genetic differentiation and loss of secondary metabolite production constitute a rapid process observed previously with laboratory strains of B. subtilis. The most widely used B. subtilis model strains (e.g., 168 and PY79) have rapidly lost their ability to produce NRPs during domestication due to a mutation in the *sfp* gene (52). The lack of surfactin production reduced swarming. Therefore, easy cultivation on agar media probably influenced the domestication of this species.

Interestingly, hampered surfactin production was noticed in isolate MB9_B4. We hypothesize that the reduction of surfactin production might be due to altered gene regulation by a mutation in the *comA* gene. However, it is unclear to what extent the ComA protein level is affected due to mutation in the translation-initiating methionine and whether the potentially altered level of this transcription factor in strain MB9_B4 mitigates sufficient binding to the promoter region of *srf* to activate its transcription (53). Repair of the *comA* mutation could corroborate whether this single mutation causes the reduced surfactin production in MB9_B4. Notably, the *srf* operon also codes for the anti-adaptor protein ComS required for competence development (54). The reduced expression of the *srf* gene cluster would also attenuate the cotranscription of *comS*, therefore causing diminished competence. However, we found no evidence that competence is affected in MB9_B4 compared to other isolates.

Besides reduced surfactin production, plipastatin was not detected in the extracts of three isolates: 73, MB9_B6, and P5_B2. A point-nonsense mutation could be identified in the *ppsB* gene of strain MB9_B6, which possibly hinders the assembly of a functional plipastatin-producing complex. In contrast, strains 73 and P5_B2 show a complete loss of *ppsB*, and only fragments of the core genes *ppsA*, *ppsC*, and *ppsD* are present, resulting in a lack of plipastatin production. Intriguingly, these two strains isolated from soil samples in Germany and Denmark carry very similar deletions in the *pps* gene cluster. Finally, strain P5_B2 lost the complete *pks* gene cluster.

These intriguing examples of partial BGCs in environmental B. subtilis strains highlight the possibility for secondary metabolite production loss in nature. Similar findings were obtained in a bioinformatic study investigating the phylogeny and distribution of BGCs among various *Bacillus* strains isolated from around the globe. The authors found fragmented BGCs of plipastatin and fengycin in several strains, highlighting the conservation of these gene losses in particular clades (27). These observations suggest either that the selection pressure is not strong enough to maintain the production of these specialized metabolites in particular niches or that secondary metabolites can be shared as common goods in bacterial populations, and these derivatives can act as cheaters. Besides, the appearance of producer and nonproducer strains in a bacterial population can also be seen as a division of labor, as suggested to be present in the *Streptomyces* genus (55). It remains to be examined whether the derivatives with mutated secondary metabolite production have altered fitness when growing in soil. The role and effect of most secondary metabolites under natural settings might differ from *in vitro* investigations and need to be further unraveled. Interestingly, the observed gene loss of B. subtilis BGCs in natural isolates suggests that it is not as adverse as expected from *in vitro* laboratory observations.

The investigation of intraspecies interactions of coisolated strains was driven by the questions of whether these isolates inhibit each other and whether a linkage to their accessory BGC predictions is observable. We could observe that coisolated strains with the same predicted accessory gene clusters showed mutually no inhibition (MB9_B1, MB9_B4, and MB9_B6; P8_B1 and P8_B3) or mutually minor inhibition (MB8_B1 and MB8_B10). In contrast, strains with different accessory BGCs showed mutual (MB8_B7 and MB8_B1; MB8_B7 and MB8_B10) and unilateral (73 and 75; P5_B1 and P5_B2) inhibition. However, minor inhibition was even caused by strain P5_B2, having no predicted accessory BGCs. The findings suggest that the presence or absence of the tested four accessory BGCs impact the intraspecies interactions. Nevertheless, some strains with the same BGC predictions showed minor inhibition, indicating that besides the predicted known secondary metabolites, unknown secondary metabolites or other genes or compounds are involved in the interactions. We concentrated in this approach only on coisolated strains, but the results indicated that the closely related coisolates (MB9_B1, MB9_B4, and MB9_B6; P8_B1 and P8_B3 [Fig. 3]) showed no inhibition of each other. Notably, the most potent interactions were observable when B. subtilis strains P8_B1 and P8_B3 were screened against B. licheniformis P8_B2. A previous study demonstrated a negative correlation between interspecies interactions or kin discrimination and phylogeny (56), which explains the strongest interaction for the least related tested strains. An extended interaction screening with all strain combinations of B. subtilis could compare the impact of relatedness and sampling site on the inhibition potential. Furthermore, it would be interesting to see if the strains produce the predicted secondary metabolites, and additional mutant-based approaches could clarify their direct impact on the intraspecies inhibition potential.

## MATERIALS AND METHODS

### Strains, media, and chemicals.

All strains that were used in this study or that were used solely as genomic DNA (gDNA) donors for transformation are listed in Table S1. For routine growth, bacterial cells were cultured in lysogeny broth medium (LB-Lennox, Carl Roth, Germany; 10 g liter^−1^ tryptone, 5 g liter^−1^ yeast extract, and 5 g liter^−1^ NaCl) supplemented with 1.5% Bacto agar if required. When necessary, the following antibiotics were used: macrolide-lincosamide-streptogramin B (MLS) antibiotics (1 μg ml^−1^ erythromycin and 25 μg ml^−1^ lincomycin), spectinomycin (100 μg ml^−1^), chloramphenicol (5 μg ml^−1^), tetracycline (10 μg ml^−1^), erythromycin (5 μg ml^−1^), and ampicillin (100 μg ml^−1^). Soil isolates were obtained from 11 sampling sites in Germany and Denmark (see Table S1 for coordinates) by selecting for sporeformers in the soil. Soil samples were mixed with 0.9% saline solution, vortexed on a rotary shaker for 2 min, incubated at 80°C for 25 min, and serially diluted on LB medium solidified with 1.5% agar (57). Highly structured colonies were targeted, and isolation of B. subtilis strains was confirmed using 16S RNA sequencing followed by whole-genome sequencing of 13 selected strains (45) and one additional isolate identified as B. licheniformis.

10.1128/mSystems.00770-20.6 TABLE S1 Bacterial strains used in this study. Download Table S1, PDF file, 0.1 MB. Copyright © 2021 Kiesewalter et al. 2021 Kiesewalter et al. https://creativecommons.org/licenses/by/4.0/ This content is distributed under the terms of the Creative Commons Attribution 4.0 International license.

F. oxysporum, F. graminearum, and *B. cinerea* were revived on potato dextrose agar (PDA; BD, USA; potato infusion at 4 g liter^−1^, glucose at 20 g liter^−1^, agar at 15 g liter^−1^) supplemented with 0.5 g liter^−1^ CuSO_4_ and 0.5 g liter^−1^ ZnSO_4_ to harvest spores.

### B. subtilis mutant strain construction.

Strains 23, 38, 39, 64, 72, 73, 75, and 77 were isolated specifically by labeling with constitutively expressed *gfp* from P_hyperspank_ using phyGFP plasmid integrated into the *amyE* locus (58). Mutant strains were obtained using natural competence (59) by transforming genomic DNA and selecting for antibiotic resistance, followed by verifying the mutation by PCR. Mutants were constructed by transforming gDNA of the following strains: *sfp* mutants from DS3337 (60), *srfAC* mutants from DS1122 (61), Δ*pksL* mutants from DS4085 (37), and Δ*ppsC* mutants from DS4114 (37). The *srfAC*-Δ*ppsC* double mutants were obtained by transforming gDNA from DS4114 (37) into the respective *srfAC* mutants.

### Antagonism assays between plant pathogenic fungi and B. subtilis soil isolates.

Spores of fungal cultures grown at 21 to 23°C for 5 to 7 days on sporulation medium were harvested using 10 ml saline-Tween solution (8 g liter^−1^ NaCl and 0.05 ml liter^−1^ Tween 80) and filtered through Miracloth (Millipore; Billerica, MA) following the protocol described by Benoit et al. (62). The spore solution was centrifuged at 5,000 rpm for 10 min, resuspended in saline-Tween solution, and stored at 4°C until use. Bacterial overnight cultures (5 μl) and fungal spore suspension were spotted on the edge (bacteria) and in the center (fungus) of PDA plates (Carl Roth, Germany; potato infusion at 4 g liter^−1^, glucose at 20 g liter^−1^, agar at 15 g liter^−1^; pH value, 5.2 ± 0.2). Plates were cultivated at 21 to 23°C for 6 days, and antagonistic observations were qualitatively documented.

### Extraction of secondary metabolites.

Bacterial strains were cultured on PDA plates and incubated at 30°C for 3 days. A 6-mm-diameter size agar plug of the bacterial culture was transferred to a 2-ml Eppendorf tube and extracted with 1 ml organic solvent (2-propanol–ethyl acetate [EtOAc] [1:3, vol/vol] containing 1% formic acid). The tubes were then sonicated for 60 min. The solutions were transferred to new tubes, evaporated under N_2_, and redissolved in 300 μl methanol (MeOH) before further sonication for 15 min, followed by 3 min of centrifugation (13,400 rpm). After centrifugation, the supernatants were transferred to clean high-performance liquid chromatography (HPLC) vials and subjected to ultrahigh-performance liquid chromatography-high resolution mass spectrometry (UHPLC-HRMS) analysis.

### UHPLC-HRMS analysis.

A volume of 1 μl extract was subjected to UHPLC-HRMS analysis. UHPLC-HRMS was performed on an Agilent Infinity 1290 UHPLC system fitted with a diode array detector. Liquid chromatography was run on an Agilent Poroshell 120 phenyl-hexyl column (2.1 by 250 mm, 2.7 μm) at 60°C with an acetonitrile (MeCN)-H_2_O gradient, both containing 20 mM formic acid. A linear gradient of 10% MeCN-H_2_O to 100% MeCN over 15 min was initially employed, followed by an isocratic condition of 100% MeCN for 2 min before returning to starting conditions of 10% MeCN-H_2_O for 3 min, all at a flow rate of 0.35 ml/min. An Agilent 6545 quadrupole time of flight (QTOF) MS equipped with an Agilent dual-jet stream electrospray ion (ESI) source was used for MS detection in positive ionization. The MS detection was performed with a drying gas temperature of 250°C, drying gas flow of 8 liters/min, sheath gas temperature of 300°C, and sheath gas flow of 12 liters/min. The capillary voltage was set to 4,000 V and nozzle voltage to 500 V. MS data were processed and analyzed using Agilent MassHunter Qualitative Analysis B.07.00.

### Intraspecies interactions.

Bacterial overnight cultures were adjusted to an optical density (OD) of 2. LB plates were prepared with the agar overlay technique: 10 ml LB medium containing 1.5% agar functioned as the bottom layer and was overlaid with 10 ml LB medium containing 1% agar preinoculated with the target strain in a 1:200 dilution. The focal strain (8 μl) was spotted onto the 25-min-predried double-layer plates and incubated at 37°C for 24 h. Interactions were evaluated by checking appearance of clearing zones between the focal colonies and bacterial lawns of the target strains.

### Bioinformatic analysis.

The genomes of 14 selected soil isolates (13 B. subtilis and 1 B. licheniformis) published by Kiesewalter et al. (45) were submitted to antiSMASH 5.0 to analyze the differences in their gene cluster predictions (46). The pan-genome pipeline Roary was applied to the Prokka annotations of the B. subtilis genomes to construct a pan-genome of genes having a 95% similarity in 99% of the isolates (63, 64). The list of present and absent genes generated by Roary was used for comparisons between selected BGCs. Single gene comparisons were conducted by aligning both the nucleotide sequences and, with seqKit (65), translated amino acid sequences with MUSCLE (66) and inspecting them in Jalview 2 (67). All gene clusters or single genes were visualized in R using the publicly available ggplot2 extensions gggenes, ggseqlogo, and ggmsa (68–71). A phylogenetic tree was calculated with FastTree 2 using the core gene alignment by Roary and visualized in R with the publicly available ggplot2 extension ggtree (72, 73).

10.1128/mSystems.00770-20.7 TABLE S2 Fungal strains used in this study. Download Table S2, PDF file, 0.05 MB. Copyright © 2021 Kiesewalter et al. 2021 Kiesewalter et al. https://creativecommons.org/licenses/by/4.0/ This content is distributed under the terms of the Creative Commons Attribution 4.0 International license.

## Supplementary Material

Reviewer comments

## References

[1] Foster KR, Bell T. 2012. Competition, not cooperation, dominates interactions among culturable microbial species. Curr Biol 22:1845–1850. doi:10.1016/j.cub.2012.08.005. 22959348

[2] Romero D, Traxler MF, López D, Kolter R. 2011. Antibiotics as signal molecules. Chem Rev 111:5492–5505. doi:10.1021/cr2000509. 21786783 PMC3173521

[3] Linares JF, Gustafsson I, Baquero F, Martinez JL. 2006. Antibiotics as intermicrobial signaling agents instead of weapons. Proc Natl Acad Sci U S A 103:19484–19489. doi:10.1073/pnas.0608949103. 17148599 PMC1682013

[4] Straight PD, Willey JM, Kolter R. 2006. Interactions between *Streptomyces coelicolor* and *Bacillus subtilis*: role of surfactants in raising aerial structures. J Bacteriol 188:4918–4925. doi:10.1128/JB.00162-06. 16788200 PMC1483000

[5] Vaz Jauri P, Bakker MG, Salomon CE, Kinkel LL. 2013. Subinhibitory antibiotic concentrations mediate nutrient use and competition among soil *Streptomyces*. PLoS One 8:e81064. doi:10.1371/journal.pone.0081064. 24339897 PMC3855208

[6] Kovács ÁT. 2019. Bacillus subtilis. Trends Microbiol 27:724–725. doi:10.1016/j.tim.2019.03.008. 31000489

[7] Caulier S, Nannan C, Gillis A, Licciardi F, Bragard C, Mahillon J. 2019. Overview of the antimicrobial compounds produced by members of the *Bacillus subtilis* group. Front Microbiol 10:302. doi:10.3389/fmicb.2019.00302. 30873135 PMC6401651

[8] Stein T. 2005. *Bacillus subtilis* antibiotics: structures, syntheses and specific functions. Mol Microbiol 56:845–857. doi:10.1111/j.1365-2958.2005.04587.x. 15853875

[9] Kaspar F, Neubauer P, Gimpel M. 2019. Bioactive secondary metabolites from *Bacillus subtilis*: a comprehensive review. J Nat Prod 82:2038–2053. doi:10.1021/acs.jnatprod.9b00110. 31287310

[10] Harwood CR, Mouillon JM, Pohl S, Arnau J. 2018. Secondary metabolite production and the safety of industrially important members of the *Bacillus subtilis* group. FEMS Microbiol Rev 42:721–738. doi:10.1093/femsre/fuy028. 30053041 PMC6199538

[11] Fan B, Blom J, Klenk H-P, Borriss R. 2017. *Bacillus amyloliquefaciens*, *Bacillus velezensis*, and *Bacillus siamensis* form an “operational group *B. amyloliquefaciens*” within the *B. subtilis* species complex. Front Microbiol 8:22. doi:10.3389/fmicb.2017.00022. 28163698 PMC5247444

[12] Ongena M, Jacques P. 2008. *Bacillus* lipopeptides: versatile weapons for plant disease biocontrol. Trends Microbiol 16:115–125. doi:10.1016/j.tim.2007.12.009. 18289856

[13] Grubbs KJ, Bleich RM, Santa Maria KC, Allen SE, Farag S, Shank EA, Bowers AA. 2017. Large-scale bioinformatics analysis of *Bacillus* genomes uncovers conserved roles of natural products in bacterial physiology. mSystems 2:e00040-17. doi:10.1128/mSystems.00040-17. 29152584 PMC5686519

[14] Quadri LEN, Weinreb PH, Lei M, Nakano MM, Zuber P, Walsh CT. 1998. Characterization of Sfp, a *Bacillus subtilis* phosphopantetheinyl transferase for peptidyl carrier protein domains in peptide synthetases. Biochemistry 37:1585–1595. doi:10.1021/bi9719861. 9484229

[15] Tsuge K, Ano T, Hirai M, Nakamura Y, Shoda M. 1999. The genes *degQ*, *pps*, and *lpa-8* (*sfp*) are responsible for conversion of *Bacillus subtilis* 168 to plipastatin production. Antimicrob Agents Chemother 43:2183–2192. doi:10.1128/AAC.43.9.2183. 10471562 PMC89444

[16] Nakano MM, Corbell N, Besson J, Zuber P. 1992. Isolation and characterization of *sfp*: a gene that functions in the production of the lipopeptide biosurfactant, surfactin, in *Bacillus subtilis*. Mol Gen Genet 232:313–321. doi:10.1007/BF00280011. 1557038

[17] Kearns DB, Losick R. 2003. Swarming motility in undomesticated *Bacillus subtilis*. Mol Microbiol 49:581–590. doi:10.1046/j.1365-2958.2003.03584.x. 12864845

[18] Grau RR, De Oña P, Kunert M, Leñini C, Gallegos-Monterrosa R, Mhatre E, Vileta D, Donato V, Hölscher T, Boland W, Kuipers OP, Kovács ÁT. 2015. A duo of potassium-responsive histidine kinases govern the multicellular destiny of *Bacillus subtilis*. mBio 6:e00581-15. doi:10.1128/mBio.00581-15. 26152584 PMC4495169

[19] Sheppard JD, Jumarie C, Cooper DG, Laprade R. 1991. Ionic channels induced by surfactin in planar lipid bilayer membranes. Biochim Biophys Acta 1064:13–23. doi:10.1016/0005-2736(91)90406-X. 1709052

[20] Heerklotz H, Wieprecht T, Seelig J. 2004. Membrane perturbation by the lipopeptide surfactin and detergents as studied by deuterium NMR. J Phys Chem B 108:4909–4915. doi:10.1021/jp0371938.

[21] Heerklotz H, Seelig J. 2007. Leakage and lysis of lipid membranes induced by the lipopeptide surfactin. Eur Biophys J 36:305–314. doi:10.1007/s00249-006-0091-5. 17051366

[22] Sabaté DC, Audisio MC. 2013. Inhibitory activity of surfactin, produced by different *Bacillus subtilis* subsp. *subtilis* strains, against *Listeria monocytogenes* sensitive and bacteriocin-resistant strains. Microbiol Res 168:125–129. doi:10.1016/j.micres.2012.11.004. 23265790

[23] Loiseau C, Schlusselhuber M, Bigot R, Bertaux J, Berjeaud JM, Verdon J. 2015. Surfactin from *Bacillus subtilis* displays an unexpected anti-*Legionella* activity. Appl Microbiol Biotechnol 99:5083–5093. doi:10.1007/s00253-014-6317-z. 25573468

[24] Gao L, Han J, Liu H, Qu X, Lu Z, Bie X. 2017. Plipastatin and surfactin coproduction by *Bacillus subtilis* pB2-L and their effects on microorganisms. Antonie Van Leeuwenhoek 110:1007–1018. doi:10.1007/s10482-017-0874-y. 28477175

[25] Arjes HA, Vo L, Dunn CM, Willis L, DeRosa CA, Fraser CL, Kearns DB, Huang KC. 2020. Biosurfactant-mediated membrane depolarization maintains viability during oxygen depletion in *Bacillus subtilis*. Curr Biol 30:1011–1022.e6. doi:10.1016/j.cub.2020.01.073. 32059765 PMC7153240

[26] Chen B, Wen J, Zhao X, Ding J, Qi G. 2020. Surfactin: a quorum-sensing signal molecule to relieve CCR in *Bacillus amyloliquefaciens*. Front Microbiol 11:631. doi:10.3389/fmicb.2020.00631. 32425896 PMC7203447

[27] Steinke K, Mohite OS, Weber T, Kovács ÁT. 2020. Phylogenetic distribution of secondary metabolites in the Bacillus subtilis species complex. bioRxiv 2020.10.28.358507. 10.1128/mSystems.00057-21 PMC8546965 33688015

[28] Umezawa H, Aoyagi T, Nishikiori T, Yamagishi Y, Okuyama A, Hamada M, Takeuchi T. 1986. Plipastatins: new inhibitors of phospholipase A2, produced by *Bacillus cereus* BMG302-fF67. J Antibiot (Tokyo) 39:737–744. doi:10.7164/antibiotics.39.737. 3089997

[29] Deleu M, Paquot M, Nylander T. 2005. Fengycin interaction with lipid monolayers at the air-aqueous interface—implications for the effect of fengycin on biological membranes. J Colloid Interface Sci 283:358–365. doi:10.1016/j.jcis.2004.09.036. 15721905

[30] Romero D, De Vicente A, Rakotoaly RH, Dufour SE, Veening JW, Arrebola E, Cazorla FM, Kuipers OP, Paquot M, Pérez-García A. 2007. The iturin and fengycin families of lipopeptides are key factors in antagonism of *Bacillus subtilis* toward *Podosphaera fusca*. Mol Plant Microbe Interact 20:430–440. doi:10.1094/MPMI-20-4-0430. 17427813

[31] Alvarez F, Castro M, Príncipe A, Borioli G, Fischer S, Mori G, Jofré E. 2012. The plant-associated *Bacillus amyloliquefaciens* strains MEP218 and ARP23 capable of producing the cyclic lipopeptides iturin or surfactin and fengycin are effective in biocontrol of sclerotinia stem rot disease. J Appl Microbiol 112:159–174. doi:10.1111/j.1365-2672.2011.05182.x. 22017648

[32] Falardeau J, Wise C, Novitsky L, Avis TJ. 2013. Ecological and mechanistic insights into the direct and indirect antimicrobial properties of *Bacillus subtilis* lipopeptides on plant pathogens. J Chem Ecol 39:869–878. doi:10.1007/s10886-013-0319-7. 23888387

[33] Roy A, Mahata D, Paul D, Korpole S, Franco OL, Mandal SM. 2013. Purification, biochemical characterization and self-assembled structure of a fengycin-like antifungal peptide from *Bacillus thuringiensis* strain SM1. Front Microbiol 4:332. doi:10.3389/fmicb.2013.00332. 24312083 PMC3836021

[34] Tang Q, Bie X, Lu Z, Lv F, Tao Y, Qu X. 2014. Effects of fengycin from *Bacillus subtilis* fmbJ on apoptosis and necrosis in *Rhizopus stolonifer*. J Microbiol 52:675–680. doi:10.1007/s12275-014-3605-3. 25098563

[35] Zhang L, Sun C. 2018. Fengycins, cyclic lipopeptides from marine *Bacillus subtilis* strains, kill the plant-pathogenic fungus *Magnaporthe grisea* by inducing reactive oxygen species production and chromatin condensation. Appl Environ Microbiol 84:e00445-18. doi:10.1128/AEM.00445-18. 29980550 PMC6122000

[36] Patel P, Huang S, Fisher S, Pirnik D, Aklonis C, Dean L, Meyers E, Fernandes P, Mayerl F. 1995. Bacillaene, a novel inhibitor of procaryotic protein synthesis produced by *Bacillus subtilis*: production, taxonomy, isolation, physico-chemical characterization and biological activity. J Antibiot (Tokyo) 48:997–1003. doi:10.7164/antibiotics.48.997. 7592068

[37] Müller S, Strack SN, Hoefler BC, Straight PD, Kearns DB, Kirby JR. 2014. Bacillaene and sporulation protect *Bacillus subtilis* from predation by *Myxococcus xanthus*. Appl Environ Microbiol 80:5603–5610. doi:10.1128/AEM.01621-14. 25002419 PMC4178607

[38] May JJ, Wendrich TM, Marahiel MA. 2001. The *dhb* operon of *Bacillus subtilis* encodes the biosynthetic template for the catecholic siderophore 2,3-dihydroxybenzoate-glycine-threonine trimeric ester bacillibactin. J Biol Chem 276:7209–7217. doi:10.1074/jbc.M009140200. 11112781

[39] Edel-Hermann V, Lecomte C. 2019. Current status of *Fusarium oxysporum formae speciales* and races. Phytopathology 109:512–530. doi:10.1094/PHYTO-08-18-0320-RVW. 30461350

[40] Chen Y, Kistler HC, Ma Z. 2019. *Fusarium graminearum* trichothecene mycotoxins: biosynthesis, regulation, and management. Annu Rev Phytopathol 57:15–39. doi:10.1146/annurev-phyto-082718-100318. 30893009

[41] Williamson B, Tudzynski B, Tudzynski P, Van Kan JAL. 2007. *Botrytis cinerea*: the cause of grey mould disease. Mol Plant Pathol 8:561–580. doi:10.1111/j.1364-3703.2007.00417.x. 20507522

[42] Abbey JA, Percival D, Abbey L, Asiedu SK, Prithiviraj B, Schilder A. 2019. Biofungicides as alternative to synthetic fungicide control of grey mould (*Botrytis cinerea*)—prospects and challenges. Biocontrol Sci Technol 29:207–228. doi:10.1080/09583157.2018.1548574.

[43] Yang H, Li X, Li X, Yu H, Shen Z. 2015. Identification of lipopeptide isoforms by MALDI-TOF-MS/MS based on the simultaneous purification of iturin, fengycin, and surfactin by RP-HPLC. Anal Bioanal Chem 407:2529–2542. doi:10.1007/s00216-015-8486-8. 25662934

[44] Ma Y, Kong Q, Qin C, Chen Y, Chen Y, Lv R, Zhou G. 2016. Identification of lipopeptides in *Bacillus megaterium* by two-step ultrafiltration and LC–ESI–MS/MS. AMB Express 6:79. doi:10.1186/s13568-016-0252-6. 27639854 PMC5026979

[45] Kiesewalter HT, Lozano-Andrade CN, Maróti G, Snyder D, Cooper VS, Jørgensen TS, Weber T, Kovács ÁT. 2020. Complete genome sequences of 13 *Bacillus subtilis* soil isolates for studying secondary metabolite diversity. Microbiol Resour Announc 9:e01406-19. doi:10.1128/MRA.01406-19. 31919181 PMC6952667

[46] Blin K, Shaw S, Steinke K, Villebro R, Ziemert N, Lee SY, Medema MH, Weber T. 2019. AntiSMASH 5.0: updates to the secondary metabolite genome mining pipeline. Nucleic Acids Res 47:W81–W87. doi:10.1093/nar/gkz310. 31032519 PMC6602434

[47] Phelan RW, Barret M, Cotter PD, O’Connor PM, Chen R, Morrissey JP, Dobson ADW, O’Gara F, Barbosa TM. 2013. Subtilomycin: a new lantibiotic from *Bacillus subtilis* strain MMA7 isolated from the marine sponge *Haliclona simulans*. Mar Drugs 11:1878–1898. doi:10.3390/md11061878. 23736764 PMC3721211

[48] Roggiani M, Dubnau D. 1993. ComA, a phosphorylated response regulator protein of *Bacillus subtilis*, binds to the promoter region of *srfA*. J Bacteriol 175:3182–3187. doi:10.1128/jb.175.10.3182-3187.1993. 8387999 PMC204641

[49] Nakano MM, Zuber P. 1989. Cloning and characterization of *srfB*, a regulatory gene involved in surfactin production and competence in *Bacillus subtilis*. J Bacteriol 171:5347–5353. doi:10.1128/jb.171.10.5347-5353.1989. 2507521 PMC210372

[50] Nakano MM, Xia L, Zuber P. 1991. Transcription initiation region of the *srfA* operon, which is controlled by the *comP*-*comA* signal transduction system in *Bacillus subtilis*. J Bacteriol 173:5487–5493. doi:10.1128/jb.173.17.5487-5493.1991. 1715856 PMC208261

[51] Wang X, Luo C, Liu Y, Nie Y, Liu Y, Zhang R, Chen Z. 2010. Three non-aspartate amino acid mutations in the ComA response regulator receiver motif severely decrease surfactin production, competence development, and spore formation in *Bacillus subtilis*. J Microbiol Biotechnol 20:301–310. doi:10.4014/jmb.0906.06025. 20208433

[52] Zeigler DR, Prágai Z, Rodriguez S, Chevreux B, Muffler A, Albert T, Bai R, Wyss M, Perkins JB. 2008. The origins of 168, W23, and other *Bacillus subtilis* legacy strains. J Bacteriol 190:6983–6995. doi:10.1128/JB.00722-08. 18723616 PMC2580678

[53] Hu F, Liu Y, Li S. 2019. Rational strain improvement for surfactin production: enhancing the yield and generating novel structures. Microb Cell Fact 18:42. doi:10.1186/s12934-019-1089-x. 30819187 PMC6394072

[54] Hamoen LW, Eshuis H, Jongbloed J, Venema G, van Sinderen D. 1995. A small gene, designated *comS*, located within the coding region of the fourth amino acid‐activation domain of *srfA*, is required for competence development in *Bacillus subtilis*. Mol Microbiol 15:55–63. doi:10.1111/j.1365-2958.1995.tb02220.x. 7752896

[55] Zhang Z, Du C, de Barsy F, Liem M, Liakopoulos A, van Wezel GP, Choi YH, Claessen D, Rozen DE. 2020. Antibiotic production in *Streptomyces* is organized by a division of labor through terminal genomic differentiation. Sci Adv 6:eaay5781. doi:10.1126/sciadv.aay5781. 31998842 PMC6962034

[56] Lyons NA, Kolter R. 2017. *Bacillus subtilis* protects public goods by extending kin discrimination to closely related species. mBio 8:e00723-17. doi:10.1128/mBio.00723-17. 28679746 PMC5573675

[57] Gallegos-Monterrosa R, Mhatre E, Kovács ÁT. 2016. Specific *Bacillus subtilis* 168 variants form biofilms on nutrient-rich medium. Microbiology (Reading) 162:1922–1932. doi:10.1099/mic.0.000371. 27655338

[58] Van Gestel J, Weissing FJ, Kuipers OP, Kovács ÁT. 2014. Density of founder cells affects spatial pattern formation and cooperation in *Bacillus subtilis* biofilms. ISME J 8:2069–2079. doi:10.1038/ismej.2014.52. 24694715 PMC4184017

[59] Anagnostopoulos C, Spizizen J. 1961. Requirements for transformation in *Bacillus subtilis*. J Bacteriol 81:741–746. doi:10.1128/JB.81.5.741-746.1961. 16561900 PMC279084

[60] Patrick JE, Kearns DB. 2009. Laboratory strains of *Bacillus subtilis* do not exhibit swarming motility. J Bacteriol 191:7129–7133. doi:10.1128/JB.00905-09. 19749039 PMC2772471

[61] Chen R, Guttenplan SB, Blair KM, Kearns DB. 2009. Role of the σD-dependent autolysins in *Bacillus subtilis* population heterogeneity. J Bacteriol 191:5775–5784. doi:10.1128/JB.00521-09. 19542270 PMC2737971

[62] Benoit I, van den Esker MH, Patyshakuliyeva A, Mattern DJ, Blei F, Zhou M, Dijksterhuis J, Brakhage AA, Kuipers OP, de Vries RP, Kovács ÁT. 2015. *Bacillus subtilis* attachment to *Aspergillus niger* hyphae results in mutually altered metabolism. Environ Microbiol 17:2099–2113. doi:10.1111/1462-2920.12564. 25040940

[63] Seemann T. 2014. Prokka: rapid prokaryotic genome annotation. Bioinformatics 30:2068–2069. doi:10.1093/bioinformatics/btu153. 24642063

[64] Page AJ, Cummins CA, Hunt M, Wong VK, Reuter S, Holden MTG, Fookes M, Falush D, Keane JA, Parkhill J. 2015. Roary: rapid large-scale prokaryote pan genome analysis. Bioinformatics 31:3691–3693. doi:10.1093/bioinformatics/btv421. 26198102 PMC4817141

[65] Shen W, Le S, Li Y, Hu F. 2016. SeqKit: a cross-platform and ultrafast toolkit for FASTA/Q file manipulation. PLoS One 11:e0163962. doi:10.1371/journal.pone.0163962. 27706213 PMC5051824

[66] Edgar RC. 2004. MUSCLE: multiple sequence alignment with high accuracy and high throughput. Nucleic Acids Res 32:1792–1797. doi:10.1093/nar/gkh340. 15034147 PMC390337

[67] Waterhouse AM, Procter JB, Martin DMA, Clamp M, Barton GJ. 2009. Jalview version 2—a multiple sequence alignment editor and analysis workbench. Bioinformatics 25:1189–1191. doi:10.1093/bioinformatics/btp033. 19151095 PMC2672624

[68] Wickham H. 2011. ggplot2. WIREs Comp Stat 3:180–185. doi:10.1002/wics.147.

[69] Wilkins D. 2019. gggenes: draw gene arrow maps in “ggplot2.” R package version 0.4.0. https://CRAN.R-project.org/package=gggenes.

[70] Wagih O. 2017. ggseqlogo: a “ggplot2” extension for drawing publication-ready sequence logos. https://CRAN.R-project.org/package=ggseqlogo.

[71] Yu G, Zhou L, Huang H. 2020. ggmsa: plot multiple sequence alignment using “ggplot2.” https://CRAN.R-project.org/package=ggmsa.

[72] Price MN, Dehal PS, Arkin AP. 2010. FastTree 2 - Approximately maximum-likelihood trees for large alignments. PLoS One 5:e9490. doi:10.1371/journal.pone.0009490. 20224823 PMC2835736

[73] Yu G. 2020. Using ggtree to visualize data on tree-like structures. Curr Protoc Bioinformatics 69:e96. doi:10.1002/cpbi.96. 32162851

