# Supplementary figures and images for: Genomic and Chemical Diversity of Bacillus subtilis Secondary Metabolites against Plant Pathogenic Fungi
Source: mSystems. 2021 Feb 23;6(1):e00770-20. doi: 10.1128/mSystems.00770-20 (PMC8573961; doi:10.1128/mSystems.00770-20)

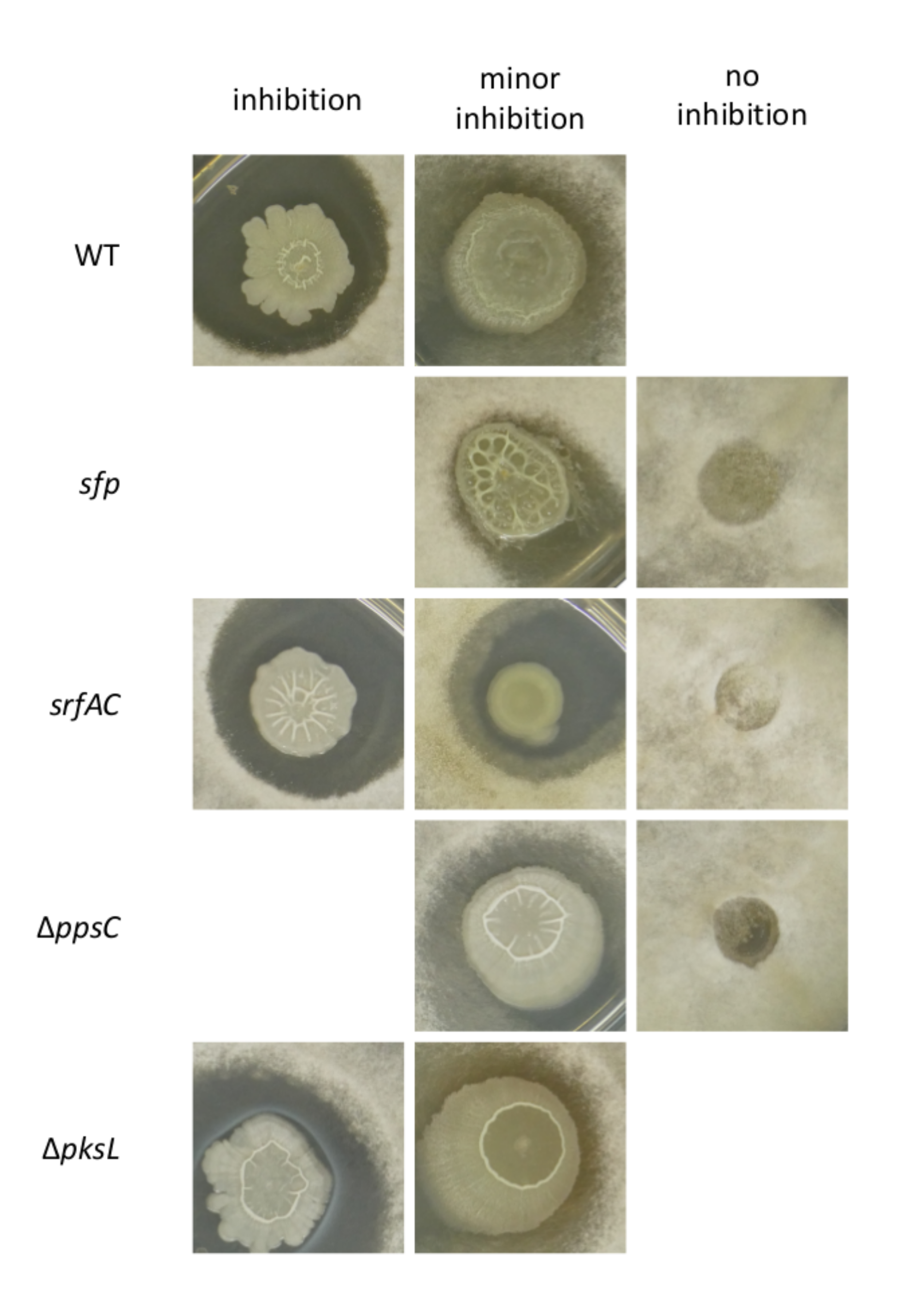

Supplement: FIG S1 [file msystems.00770-20-sf001.tif]

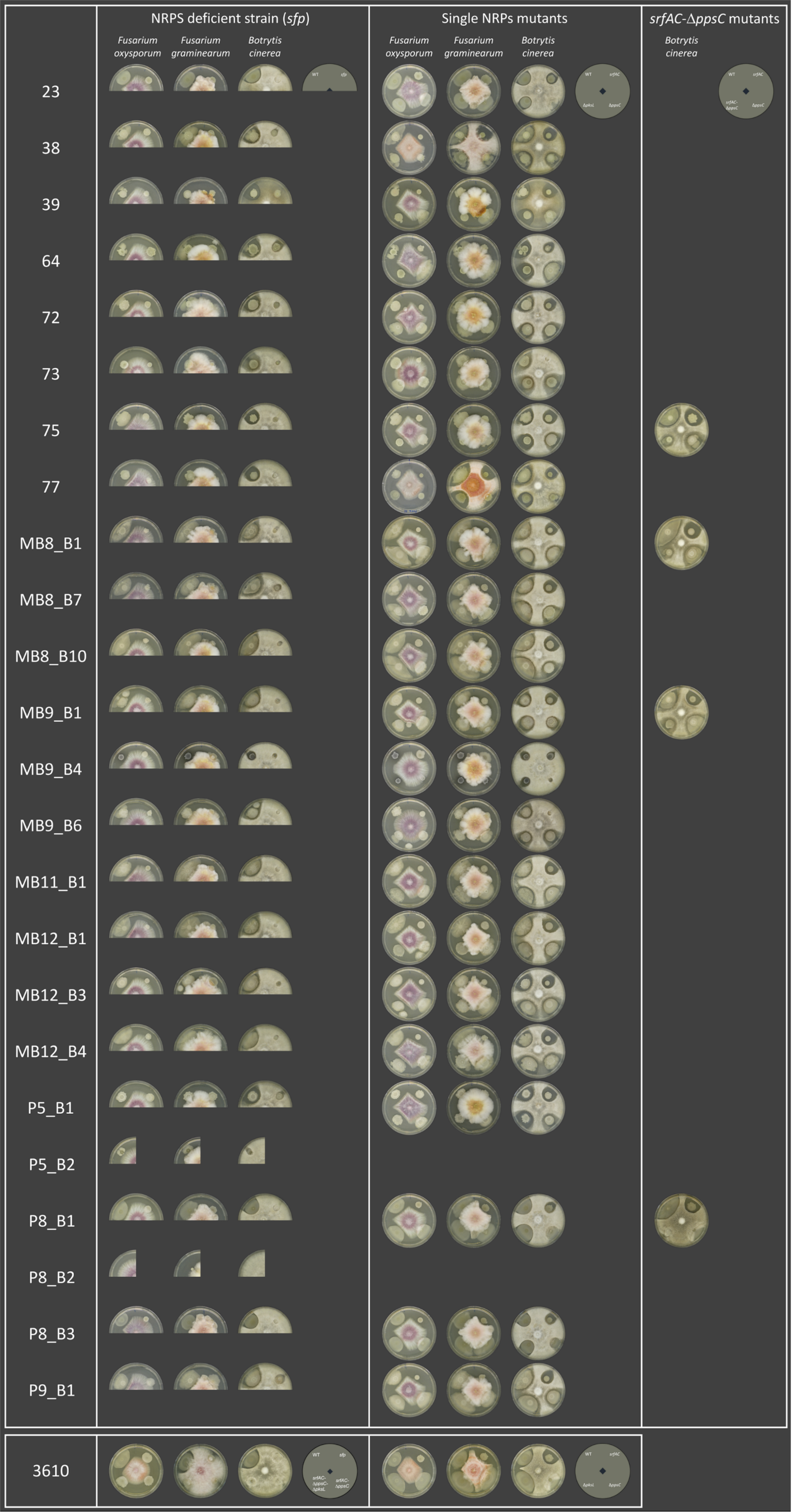

Supplement: FIG S2 [file msystems.00770-20-sf002.tif]

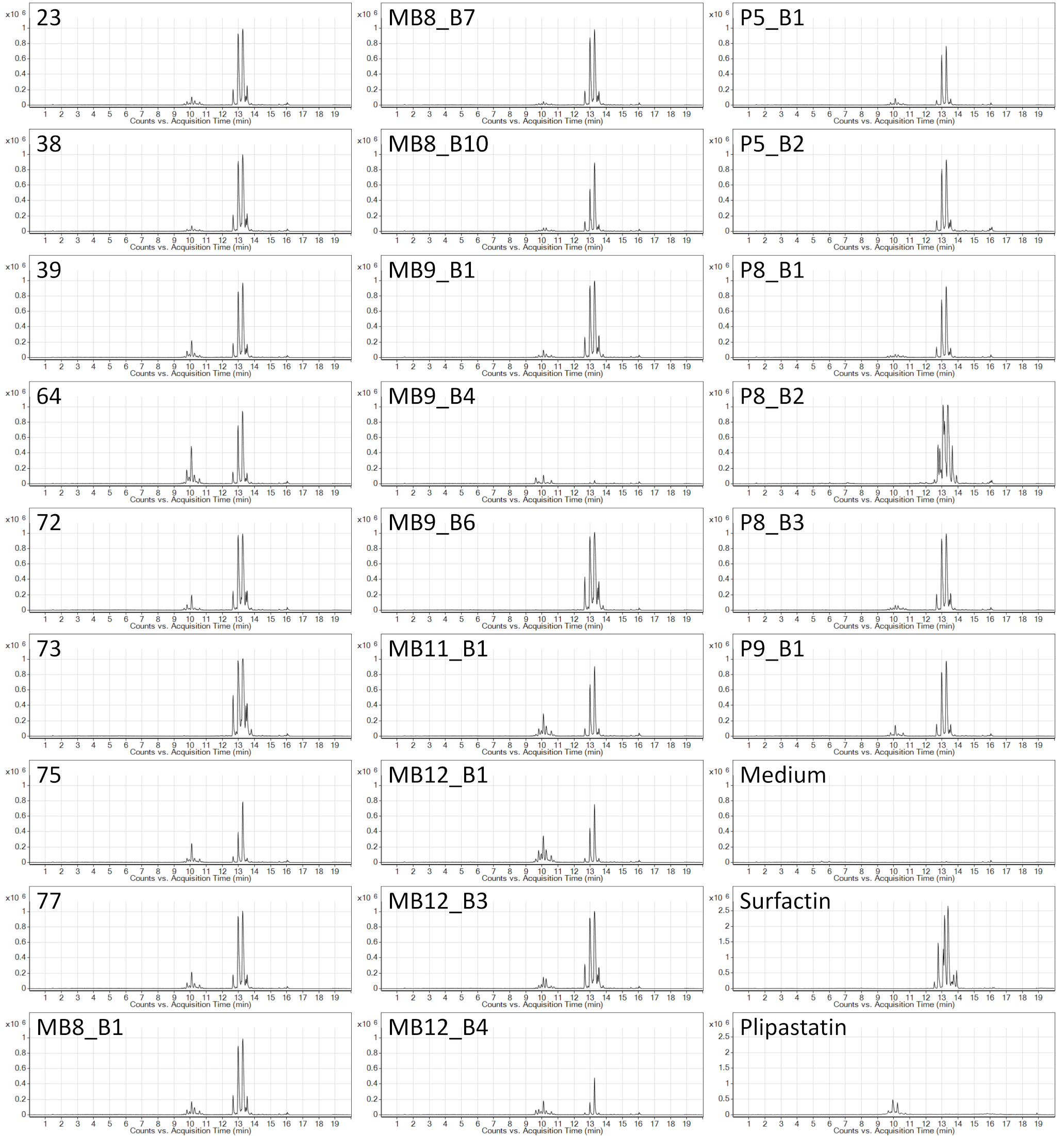

Supplement: FIG S3 [file msystems.00770-20-sf003.tif]

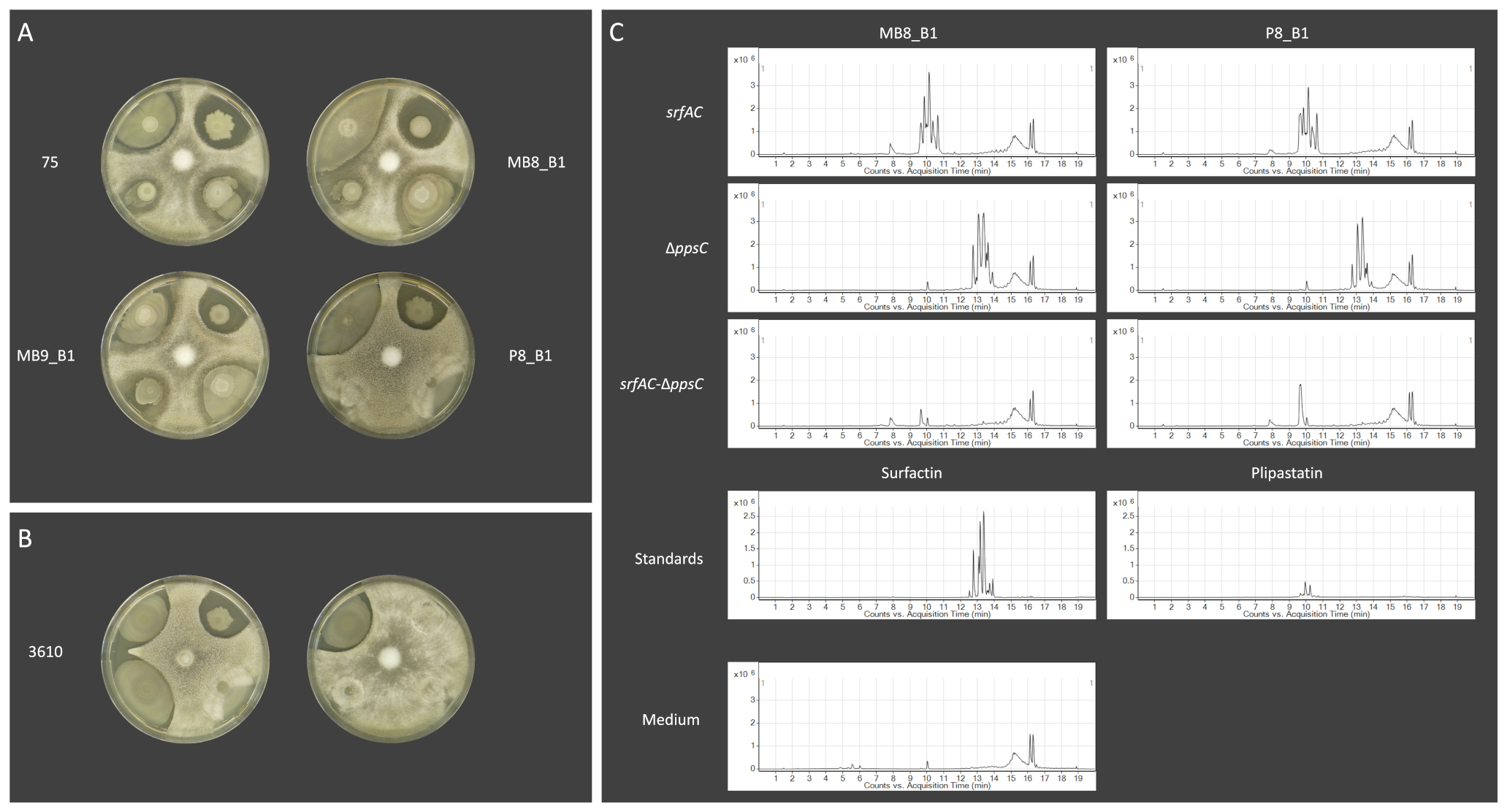

Supplement: FIG S4 [file msystems.00770-20-sf004.tif]

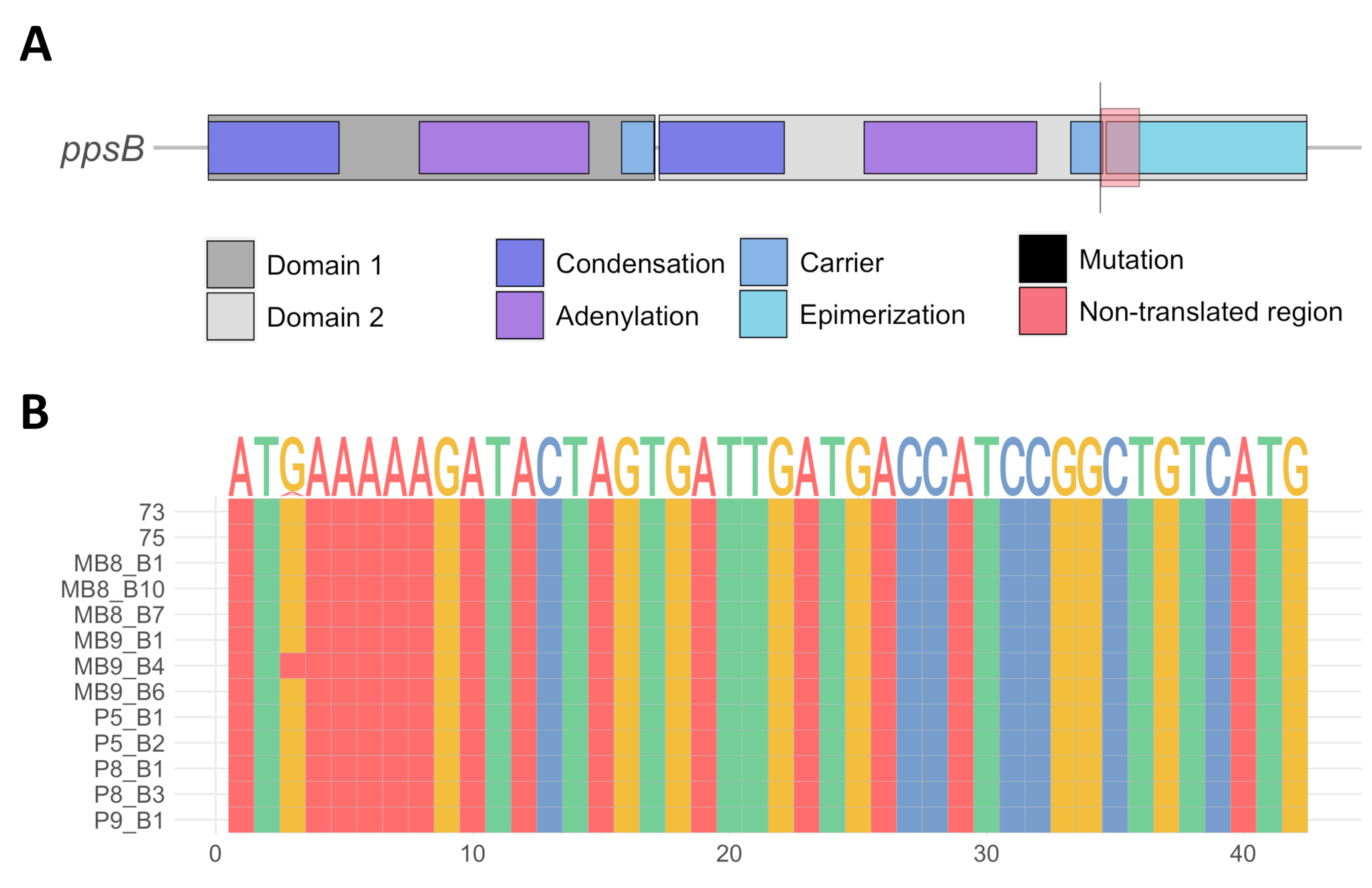

Supplement: FIG S5 [file msystems.00770-20-sf005.tif]
