# Supplementary material for: Genomic and Chemical Diversity of Bacillus subtilis Secondary Metabolites against Plant Pathogenic Fungi
Source: mSystems. 2021 Feb 23;6(1):e00770-20. doi: 10.1128/mSystems.00770-20 (PMC8573961; doi:10.1128/mSystems.00770-20)
Supplement: TABLE S1 [file msystems.00770-20-st001.pdf]

Table S1. Bacterial strains used in this study

Strains	Characteristics	Reference
NCIB3610	WT/Undomesticated strain	Lab stock
DS3337	3610 *sfp*::mls	(1)
DS1122	3610 *srfAC*::Tn*10* (Spec^R)	(2)
DS4085	3610 Δ*pksL* (Chl^R)	(3)
DS4114	3610 Δ*ppsC* (Tet^R)	(3)
MB8_B1	*B. subtilis* soil isolate from sampling site 55.843861, 12.424770	(4)
MB8_B7	*B. subtilis* soil isolate from sampling site 55.843861, 12.424770	This study
MB8_B10	*B. subtilis* soil isolate from sampling site 55.843861, 12.424770	This study
MB9_B1	*B. subtilis* soil isolate from sampling site 55.843861, 12.424770	(4)
MB9_B4	*B. subtilis* soil isolate from sampling site 55.843861, 12.424770	This study
MB9_B6	*B. subtilis* soil isolate from sampling site 55.843861, 12.424770	This study
MB11_B1	*B. subtilis* soil isolate from sampling site 55.843861, 12.424770	This study
MB12_B1	*B. subtilis* soil isolate from sampling site 55.843861, 12.424770	This study
MB12_B3	*B. subtilis* soil isolate from sampling site 55.843861, 12.424770	This study
MB12_B4	*B. subtilis* soil isolate from sampling site 55.843861, 12.424770	This study
P5_B1	*B. subtilis* soil isolate from sampling site 55.788800, 12.558300	(4)
P5_B2	*B. subtilis* soil isolate from sampling site 55.788800, 12.558300	This study
P8_B1	*B. subtilis* soil isolate from sampling site 55.795200, 12.580600	(4)
P8_B2	*B. licheniformis* soil isolate from sampling site 55.795200, 12.580600	This study
P8_B3	*B. subtilis* soil isolate from sampling site 55.795200, 12.580600	This study
P9_B1	*B. subtilis* soil isolate from sampling site 55.791200, 12.575100	(4)
23	*B. subtilis* soil isolate from sampling site 50.718551, 10.951691	This study
38	*B. subtilis* soil isolate from sampling site 50.731996, 10.914328	This study
39	*B. subtilis* soil isolate from sampling site 50.731996, 10.914328	This study
64	*B. subtilis* soil isolate from sampling site 50.729170, 10.924770	This study
72	*B. subtilis* soil isolate from sampling site 50.725876, 10.916218	This study
73	*B. subtilis* soil isolate from sampling site 50.725876, 10.916218	This study
75	*B. subtilis* soil isolate from sampling site 50.725876, 10.916218	(4)
77	*B. subtilis* soil isolate from sampling site 50.725876, 10.916218	This study
DTUB44	MB8_B1 *sfp*::mls	This study
DTUB45	MB8_B7 *sfp*::mls	This study
DTUB46	MB8_B10 *sfp*::mls	This study
DTUB47	MB9_B1 *sfp*::mls	This study
DTUB48	MB9_B4 *sfp*::mls	This study
DTUB49	MB9_B6 *sfp*::mls	This study
DTUB50	MB11_B1 *sfp*::mls	This study
DTUB51	MB12_B1 *sfp*::mls	This study
DTUB52	MB12_B3 *sfp*::mls	This study
DTUB53	MB12_B4 *sfp*::mls	This study
DTUB55	P5_B1 *sfp*::mls	This study
DTUB56	P5_B2 *sfp*::mls	This study
DTUB57	P8_B1 *sfp*::mls	This study
DTUB58	P8_B3 *sfp*::mls	This study
DTUB59	P9_B1 *sfp*::mls	This study
DTUB60	23 *amyE*::$P_{hyperspank}$-*gfp* (Chl^R); *sfp*::mls	This study
DTUB61	38 *amyE*::$P_{hyperspank}$-*gfp* (Chl^R); *sfp*:: mls	This study

DTUB62	39 *amyE*::$P_{hyperspank}$-*gfp* (Chl^R); *sfp*:: mls	This study
DTUB63	64 *amyE*::$P_{hyperspank}$-*gfp* (Chl^R); *sfp*:: mls	This study
DTUB64	72 *amyE*::$P_{hyperspank}$-*gfp* (Chl^R); *sfp*:: mls	This study
DTUB65	73 *amyE*::$P_{hyperspank}$-*gfp* (Chl^R); *sfp*:: mls	This study
DTUB66	75 *amyE*::$P_{hyperspank}$-*gfp* (Chl^R); *sfp*:: mls	This study
DTUB67	77 *amyE*::$P_{hyperspank}$-*gfp* (Chl^R); *sfp*:: mls	This study
DTUB68	MB8_B1 *srfAC*::Tn*10* ($Spec^R$)	(4)
DTUB69	MB8_B7 *srfAC*::Tn*10* ($Spec^R$)	This study
DTUB70	MB8_B10 *srfAC*::Tn*10* ($Spec^R$)	This study
DTUB71	MB9_B1 *srfAC*::Tn*10* ($Spec^R$)	(4)
DTUB72	MB9_B4 *srfAC*::Tn*10* ($Spec^R$)	This study
DTUB73	MB9_B6 *srfAC*::Tn*10* ($Spec^R$)	This study
DTUB74	MB11_B1 *srfAC*::Tn*10* ($Spec^R$)	This study
DTUB75	MB12_B1 *srfAC*::Tn*10* ($Spec^R$)	This study
DTUB76	MB12_B3 *srfAC*::Tn*10* ($Spec^R$)	This study
DTUB77	MB12_B4 *srfAC*::Tn*10* ($Spec^R$)	This study
DTUB79	P5_B1 *srfAC*::Tn*10* ($Spec^R$)	(4)
DTUB80	P8_B1 *srfAC*::Tn*10* ($Spec^R$)	(4)
DTUB81	P8_B3 *srfAC*::Tn*10* ($Spec^R$)	This study
DTUB82	P9_B1 *srfAC*::Tn*10* ($Spec^R$)	(4)
DTUB83	23 *amyE*::$P_{hyperspank}$-*gfp* (Chl^R); *srfAC*::Tn*10* ($Spec^R$)	This study
DTUB84	38 *amyE*::$P_{hyperspank}$-*gfp* (Chl^R); *srfAC*::Tn*10* ($Spec^R$)	This study
DTUB85	39 *amyE*::$P_{hyperspank}$-*gfp* (Chl^R); *srfAC*::Tn*10* ($Spec^R$)	This study
DTUB86	64 *amyE*::$P_{hyperspank}$-*gfp* (Chl^R); *srfAC*::Tn*10* ($Spec^R$)	This study
DTUB87	72 *amyE*::$P_{hyperspank}$-*gfp* (Chl^R); *srfAC*::Tn*10* ($Spec^R$)	This study
DTUB88	73 *amyE*::$P_{hyperspank}$-*gfp* (Chl^R); *srfAC*::Tn*10* ($Spec^R$)	This study
DTUB89	75 *amyE*::$P_{hyperspank}$-*gfp* (Chl^R); *srfAC*::Tn*10* ($Spec^R$)	(4)
DTUB90	77 *amyE*::$P_{hyperspank}$-*gfp* (Chl^R); *srfAC*::Tn*10* ($Spec^R$)	This study
DTUB91	MB8_B1 Δ*pksL* (Chl^R)	This study
DTUB92	MB8_B7 Δ*pksL* (Chl^R)	This study
DTUB93	MB8_B10 Δ*pksL* (Chl^R)	This study
DTUB94	MB9_B1 Δ*pksL* (Chl^R)	This study
DTUB95	MB9_B4 Δ*pksL* (Chl^R)	This study
DTUB96	MB9_B6 Δ*pksL* (Chl^R)	This study
DTUB97	MB11_B1 Δ*pksL* (Chl^R)	This study
DTUB98	MB12_B1 Δ*pksL* (Chl^R)	This study
DTUB99	MB12_B3 Δ*pksL* (Chl^R)	This study
DTUB100	MB12_B4 Δ*pksL* (Chl^R)	This study
DTUB102	P5_B1 Δ*pksL* (Chl^R)	This study
DTUB103	P8_B1 Δ*pksL* (Chl^R)	This study
DTUB104	P8_B3 Δ*pksL* (Chl^R)	This study
DTUB105	P9_B1 Δ*pksL* (Chl^R)	This study
DTUB106	23 *amyE*::$P_{hyperspank}$-*gfp* (Chl^R); Δ*pksL* (Ery^R)	This study
DTUB107	38 *amyE*::$P_{hyperspank}$-*gfp* (Chl^R); Δ*pksL* (Ery^R)	This study
DTUB108	39 *amyE*::$P_{hyperspank}$-*gfp* (Chl^R); Δ*pksL* (Ery^R)	This study
DTUB109	64 *amyE*::$P_{hyperspank}$-*gfp* (Chl^R); Δ*pksL* (Ery^R)	This study
DTUB110	72 *amyE*::$P_{hyperspank}$-*gfp* (Chl^R); Δ*pksL* (Ery^R)	This study
DTUB111	73 *amyE*::$P_{hyperspank}$-*gfp* (Chl^R); Δ*pksL* (Ery^R)	This study
DTUB112	75 *amyE*::$P_{hyperspank}$-*gfp* (Chl^R); Δ*pksL* (Ery^R)	This study

DTUB113	77 *amyE*::$P_{hyperspank}$-*gfp* (Chl^R); Δ*pksL* (Ery^R)	This study
DTUB114	MB8_B1 Δ*ppsC* (Tet^R)	This study
DTUB115	MB8_B7 Δ*ppsC* (Tet^R)	This study
DTUB116	MB8_B10 Δ*ppsC* (Tet^R)	This study
DTUB117	MB9_B1 Δ*ppsC* (Tet^R)	This study
DTUB118	MB9_B4 Δ*ppsC* (Tet^R)	This study
DTUB119	MB9_B6 Δ*ppsC* (Tet^R)	This study
DTUB120	MB11_B1 Δ*ppsC* (Tet^R)	This study
DTUB121	MB12_B1 Δ*ppsC* (Tet^R)	This study
DTUB122	MB12_B3 Δ*ppsC* (Tet^R)	This study
DTUB123	MB12_B4 Δ*ppsC* (Tet^R)	This study
DTUB125	P5_B1 Δ*ppsC* (Tet^R)	This study
DTUB126	P8_B1 Δ*ppsC* (Tet^R)	This study
DTUB127	P8_B3 Δ*ppsC* (Tet^R)	This study
DTUB128	P9_B1 Δ*ppsC* (Tet^R)	This study
DTUB129	23 *amyE*::$P_{hyperspank}$-*gfp* (Chl^R); Δ*ppsC* (Tet^R)	This study
DTUB130	38 *amyE*::$P_{hyperspank}$-*gfp* (Chl^R); Δ*ppsC* (Tet^R)	This study
DTUB131	39 *amyE*::$P_{hyperspank}$-*gfp* (Chl^R); Δ*ppsC* (Tet^R)	This study
DTUB132	64 *amyE*::$P_{hyperspank}$-*gfp* (Chl^R); Δ*ppsC* (Tet^R)	This study
DTUB133	72 *amyE*::$P_{hyperspank}$-*gfp* (Chl^R); Δ*ppsC* (Tet^R)	This study
DTUB134	73 *amyE*::$P_{hyperspank}$-*gfp* (Chl^R); Δ*ppsC* (Tet^R)	This study
DTUB135	75 *amyE*::$P_{hyperspank}$-*gfp* (Chl^R); Δ*ppsC* (Tet^R)	This study
DTUB136	77 *amyE*::$P_{hyperspank}$-*gfp* (Chl^R); Δ*ppsC* (Tet^R)	This study
DTUB142	MB8_B1 *srfAC*::Tn*10* ($Spec^R$); Δ*ppsC* (Tet^R)	This study
DTUB143	MB9_B1 *srfAC*::Tn*10* ($Spec^R$); Δ*ppsC* (Tet^R)	This study
DTUB144	P8_B1 *srfAC*::Tn*10* ($Spec^R$); Δ*ppsC* (Tet^R)	This study
DTUB145	75 *amyE*::$P_{hyperspank}$-*gfp* (Chl^R); *srfAC*::Tn*10* ($Spec^R$); Δ*ppsC* (Tet^R)	This study

REFERENCES

1. Patrick JE, Kearns DB. 2009. Laboratory strains of Bacillus subtilis do not exhibit swarming motility. J Bacteriol 191:7129–7133.
2. Chen R, Guttenplan SB, Blair KM, Kearns DB. 2009. Role of the σD-dependent autolysins in Bacillus subtilis population heterogeneity. J Bacteriol 191:5775–5784.
3. Müller S, Strack SN, Hoefler BC, Straight PD, Kearns DB, Kirby JR. 2014. Bacillaene and sporulation protect bacillus subtilis from predation by myxococcus xanthus. Appl Environ Microbiol 80:5603–5610.
4. Thérien M, Kiesewalter HT, Auria E, Charron-Lamoureux V, Wibowo M, Maróti G, Kovács ÁT, Beauregard PB. 2020. Surfactin production is not essential for pellicle and root-associated biofilm development of Bacillus subtilis. Biofilm 2:100021.
