# Supplementary material for: Genomic and Chemical Diversity of Bacillus subtilis Secondary Metabolites against Plant Pathogenic Fungi
Source: mSystems. 2021 Feb 23;6(1):e00770-20. doi: 10.1128/mSystems.00770-20 (PMC8573961; doi:10.1128/mSystems.00770-20)
Supplement: TABLE S2 [file msystems.00770-20-st002.pdf]

Table S2. Fungal strains used in this study

Fungal species	Isolation	Source
Fusarium oxysporum IBT 40872	From surface of an equipment in a Danish factory, 2005, by Anne Svendsen	IBT Culture Collection of Fungi, DTU
Fusarium graminearum IBT 41925	From malting barley, Schackenborg, Denmark, 2012, by Ulf Thrane	IBT Culture Collection of Fungi, DTU
Botrytis cinerea IBT 42565	From indoor air in a Kitchen, Kongens Lyngby, Denmark, 2018, by Birgitte Andersen	IBT Culture Collection of Fungi, DTU
